# Bioinformatics Analysis of Gut Microbiota and CNS Transcriptome in Virus-Induced Acute Myelitis and Chronic Inflammatory Demyelination; Potential Association of Distinct Bacteria With CNS IgA Upregulation

**DOI:** 10.3389/fimmu.2020.01138

**Published:** 2020-07-07

**Authors:** Seiichi Omura, Fumitaka Sato, Ah-Mee Park, Mitsugu Fujita, Sundar Khadka, Yumina Nakamura, Aoshi Katsuki, Kazuto Nishio, Felicity N. E. Gavins, Ikuo Tsunoda

**Affiliations:** ^1^Department of Microbiology, Kindai University Faculty of Medicine, Osaka, Japan; ^2^Department of Microbiology and Immunology, Louisiana State University Health Sciences Center-Shreveport, Shreveport, LA, United States; ^3^Department of Genome Biology, Kindai University Faculty of Medicine, Osaka, Japan; ^4^Department of Molecular and Cellular Physiology, Louisiana State University Health Sciences Center-Shreveport, Shreveport, LA, United States; ^5^Department of Biosciences, College of Health and Life Sciences, Brunel University London, Uxbridge, United Kingdom

**Keywords:** fecal microbiome, dysbiosis, viral model for multiple sclerosis, pattern matching, predictive metagenome analysis, PICRUSt, RNA-Seq, gene expression profiles

## Abstract

Virus infections have been associated with acute and chronic inflammatory central nervous system (CNS) diseases, e.g., acute flaccid myelitis (AFM) and multiple sclerosis (MS), where animal models support the pathogenic roles of viruses. In the spinal cord, Theiler's murine encephalomyelitis virus (TMEV) induces an AFM-like disease with gray matter inflammation during the acute phase, 1 week post infection (p.i.), and an MS-like disease with white matter inflammation during the chronic phase, 1 month p.i. Although gut microbiota has been proposed to affect immune responses contributing to pathological conditions in remote organs, including the brain pathophysiology, its precise role in neuroinflammatory diseases is unclear. We infected SJL/J mice with TMEV; harvested feces and spinal cords on days 4 (before onset), 7 (acute phase), and 35 (chronic phase) p.i.; and examined fecal microbiota by 16S rRNA sequencing and CNS transcriptome by RNA sequencing. Although TMEV infection neither decreased microbial diversity nor changed overall microbiome patterns, it increased abundance of individual bacterial genera *Marvinbryantia* on days 7 and 35 p.i. and *Coprococcus* on day 35 p.i., whose pattern-matching with CNS transcriptome showed strong correlations: *Marvinbryantia* with eight T-cell receptor (TCR) genes on day 7 and with seven immunoglobulin (Ig) genes on day 35 p.i.; and *Coprococcus* with gene expressions of not only TCRs and IgG/IgA, but also major histocompatibility complex (MHC) and complements. The high gene expression of IgA, a component of mucosal immunity, in the CNS was unexpected. However, we observed substantial IgA positive cells and deposition in the CNS, as well as a strong correlation between CNS IgA gene expression and serum anti-TMEV IgA titers. Here, changes in a small number of distinct gut bacteria, but not overall gut microbiota, could affect acute and chronic immune responses, causing AFM- and MS-like lesions in the CNS. Alternatively, activated immune responses would alter the composition of gut microbiota.

## Introduction

Virus infections can induce tissue damage either by direct virus replication/killing of infected cells (viral pathology) or by immune-mediated tissue damage (immunopathology) (1, 2). Virus infections have been associated with acute and chronic inflammatory central nervous system (CNS) diseases, including acute flaccid myelitis (AFM) and multiple sclerosis (MS) (3, 4). Although inflammation of the spinal cord has been observed in both AFM and MS, lesions localize in the gray matter in AFM and in the white matter in MS.

Recently, there have been increased reports of patients with AFM, a disease of acute limb weakness, with magnetic resonance imaging (MRI) abnormalities in the gray matter of the spinal cord (5) and pleocytosis in the cerebrospinal fluid (CSF) (3, 6). AFM has been associated with infections of viruses that belong to the family *Picornaviridae*, including enteroviruses (4, 6, 7). We do not know the precise pathomechanism of human AFM, partly because there are few autopsy reports (8). Although the establishment of mouse models for AFM, using enterovirus D68 (EV-D68), has been reported (5, 9–11), the models may not replicate the human disease, because of the usage of neonatal/suckling mice and non-natural pathogen of mice. In addition, although viral pathology appears to cause CNS damage in the EV-D68 model, the different susceptibility to the EV-D68 model depending on mouse strains (11) as well as rare isolation of virus from the CSF in human AFM (4, 6) suggested a role of host factors including immunopathology in AFM (12).

MS is a chronic inflammatory demyelinating disease in the CNS, involving mainly the white matter of multiple regions, including the optic nerve, the cerebrum, and the spinal cord (13, 14). Although the precise etiology of MS remains unknown, autoimmune responses and environmental factors, particularly virus infections, have been associated with the pathogenesis of MS; autoimmune and viral etiologies have been supported by their animal models experimentally (15).

Gut microbiota has been shown to interact with the immune system. When the activation of the immune system is appropriate, this contributes to the elimination/regulation of microbes. Dysbiosis, an altered state of bacterial community, has been associated with health conditions and diseases (16, 17). Dysbiosis could lead to excessive activation of the immune system; uncontrolled immune responses can cause immunopathology, particularly in the gastrointestinal tract. For example, inflammatory bowel diseases (IBD), including ulcerative colitis and Crohn's disease, are considered to reflect inappropriate interactions between microbes and the host (18, 19). Dysbiosis has also been suggested to affect distant anatomical sites, including the CNS, which modulates CNS diseases (20).

Although dysbiosis can affect AFM by changing systemic and/or mucosal immune responses, in theory, the precise role of gut microbiota in AFM is currently unknown. On the other hand, changes of gut microbiota have been reported in MS patients, in which uncontrolled T-cell and antibody responses enhanced by dysbiosis may exacerbate CNS inflammation (13, 20, 21). However, we do not know precisely what microbial changes are associated with the uncontrolled immune responses in MS.

Theiler's murine encephalomyelitis virus (TMEV) is a natural enteric pathogen of mice and belongs to the family *Picornaviridae* (22). Experimentally, TMEV infection induces a biphasic disease: an AFM-like disease with gray matter inflammation during the acute phase, about 1 week post infection (p.i.), and an MS-like disease with white matter inflammation, which is confined in the spinal cord, during the chronic phase, 1 month p.i. During both acute and chronic phases of TMEV infection, inflammatory cells mainly composed of T-cells and macrophages have been observed in the spinal cords (23) with upregulation of adhesion molecules on inflammatory cells and blood vessels (24, 25). Immunologically, T-cell and antibody responses have been shown to play a beneficial anti-viral role during the acute phase, but play a detrimental role that induces immunopathology during the chronic phase (26, 27).

The TMEV model is a unique experimental system where one can examine how one single pathogen can induce two distinct lesions in the spinal cord: gray matter inflammation (poliomyelitis) and white matter inflammatory demyelination. Although the latter has been extensively used as a viral model for MS, the former has not been studied, despite being once used as a mouse model for poliomyelitis in the 1940s (28–30). In this study, we hypothesized that dysbiosis would be associated with acute and chronic inflammation in the spinal cord induced by TMEV. By comparing and contrasting AFM- and MS-like diseases induced by a single natural pathogen of mice, TMEV, we investigated the interactions between altered microbiome and CNS transcriptome, which would give an insight into the pathophysiology of AFM and MS.

We examined fecal microbiome and CNS transcriptome during the acute phase (day 7) and chronic phase (day 35) in TMEV infection. Although TMEV infection neither increased microbial diversities nor resulted in distinct microbiome patterns, it increased the genus *Marvinbryantia* on days 7 and 35 and the genus *Coprococcus* on day 35. The abundance of genus *Marvinbryantia* was correlated with eight T-cell receptor (TCR) genes on day 7 and with seven immunoglobulin (Ig) genes on day 35. On day 35, abundance of the genus *Coprococcus* was also correlated with gene expressions of major histocompatibility complex (MHC) and complements as well as TCRs, IgG isotypes, and IgA, which were distinct from the genes identified with the genus *Marvinbryantia*. Although the high gene expression of IgA, a component of mucosal immunity, in the CNS was unexpected, substantial IgA positive cells and IgA deposition were observed in the spinal cord. IgA gene expression was correlated with serum anti-TMEV IgA titers, although we found no cross-reactivity between TMEV and *Coprococcus* antigens. This is the first report suggesting that acute myelitis and chronic neuroinflammation with IgA responses could be influenced by the changes in bacterial abundance in a limited number of bacteria, but not overall gut microbiota changes or dysbiosis. However, one must not rule out an alternative scenario in which the activated immune responses themselves alter the composition of gut microbiota.

## Materials and Methods

### Ethics Statement

All animal experiments were approved by the Louisiana State University Health Sciences Center-Shreveport (LSUHSC-S, LA) and the Kindai University Faculty of Medicine (Osaka, Japan) Institutional Animal Care and Use Committee (IACUC) guidelines and followed the National Research Council's Guide for the Care and Use of Laboratory Animals, the Institute of Laboratory Animal Resources (ILAR), and the guideline “Fundamental Guidelines for Proper Conduct of Animal Experiment and Related Activities in Academic Research Institutions” from the Ministry of Education, Culture, Sports, Science and Technology, Japan.

### Animal Experiments

Female 5-week-old SJL/J mice (Jackson Laboratory, Bar Harbor, ME) were inoculated intracerebrally (i.c.) with 2 × 10^5^ plaque-forming units (PFU) of the Daniels (DA) strain of TMEV (31). Control mice were injected i.c. with phosphate-buffered saline (PBS). On days 4, 7, and 35 after i.c. injection, we harvested spinal cords and feces from TMEV-infected and control mice (*n* = 5 per group at each time point, between 8:00 a.m. and 10:00 a.m.). Since TMEV can be transmitted by the fecal–oral route, control and TMEV-infected mice were not co-housed. We determined the mouse number per group by power analysis (32, 33) as well as the published guidelines (34, 35) and IACUC protocols of Kindai University and LSUHSC-S. All mice were maintained under specific pathogen-free conditions in our animal care facility at LSUHSC-S or Kindai University.

### RNA Sequencing

We extracted total RNA from spinal cords by the RNeasy Mini Kit (Qiagen, Valencia, CA), according to the manufacturer's instructions. DNase treatment was conducted with the RNase-Free DNase Set (Qiagen). All samples were purified to an absorbance ratio (A260/A280) between 1.9 and 2.1.

We conducted RNA sequencing and data processing as described previously (24). Briefly, we processed 100 ng of total RNA to mRNA library and sequenced on the Illumina NextSeq™ 500 system (Illumina, San Diego, CA), according to the manufacturer's instructions. Raw sequence data were mapped on reference genome and counted. The read count data were normalized with DESeq2 package in R version 3.6.0 (36, 37). Transcriptome data have been deposited into the Gene Expression Omnibus (GEO) at the National Center for Biotechnology Information (NCBI, Bethesda, MD; accession no. GSE120041, https://www.ncbi.nlm.nih.gov/geo/query/acc.cgiacc=GSE120041).

### 16S rRNA Sequencing

We extracted DNA from feces of TMEV-infected and control mice, using the QIAamp® Fast DNA Stool Mini Kit (Qiagen), according to the manufacturer's instructions. DNA was amplified by PCR, using a primer set for V3 and V4 region of 16S rRNA. The primer sequences are as follows: forward primer = 5′-TCGTCGGCAGCGTCAGATGTGTATAAGAGACAGCCTACGGGNGGCWGCAG-3′; reverse primer = 5′-GTCTCGTGGGCTCGGAGATGTGTATAAGAGACAGGACTACHVGGGTATCTAATCC-3′. Amplified DNA samples were purified and attached with dual indices and sequencing adapters, using the Nextera® XT Index Kit (Illumina). DNA libraries were validated by the 2100 Bioanalyzer DNA 100 chip (Agilent Technologies, Santa Clara, CA), quantified by the Quant-iT^TM^ Picogreen® dsDNA Assay Kit (Thermo Fisher Scientific, Waltham, MA), and sequenced by the MiSeq® System (Illumina). Raw sequence data were denoised, demultiplexed, aligned, and visualized by QIIME 2 (38). Microbiome data have been deposited into the Sequence Read Archive (SRA) at NCBI (Bioproject accession no. PRJNA561088; Biosample accession no. SAMN12607114-SAMN12607143).

### Bioinformatics Analyses

#### Heat Map

**Figure 1:**
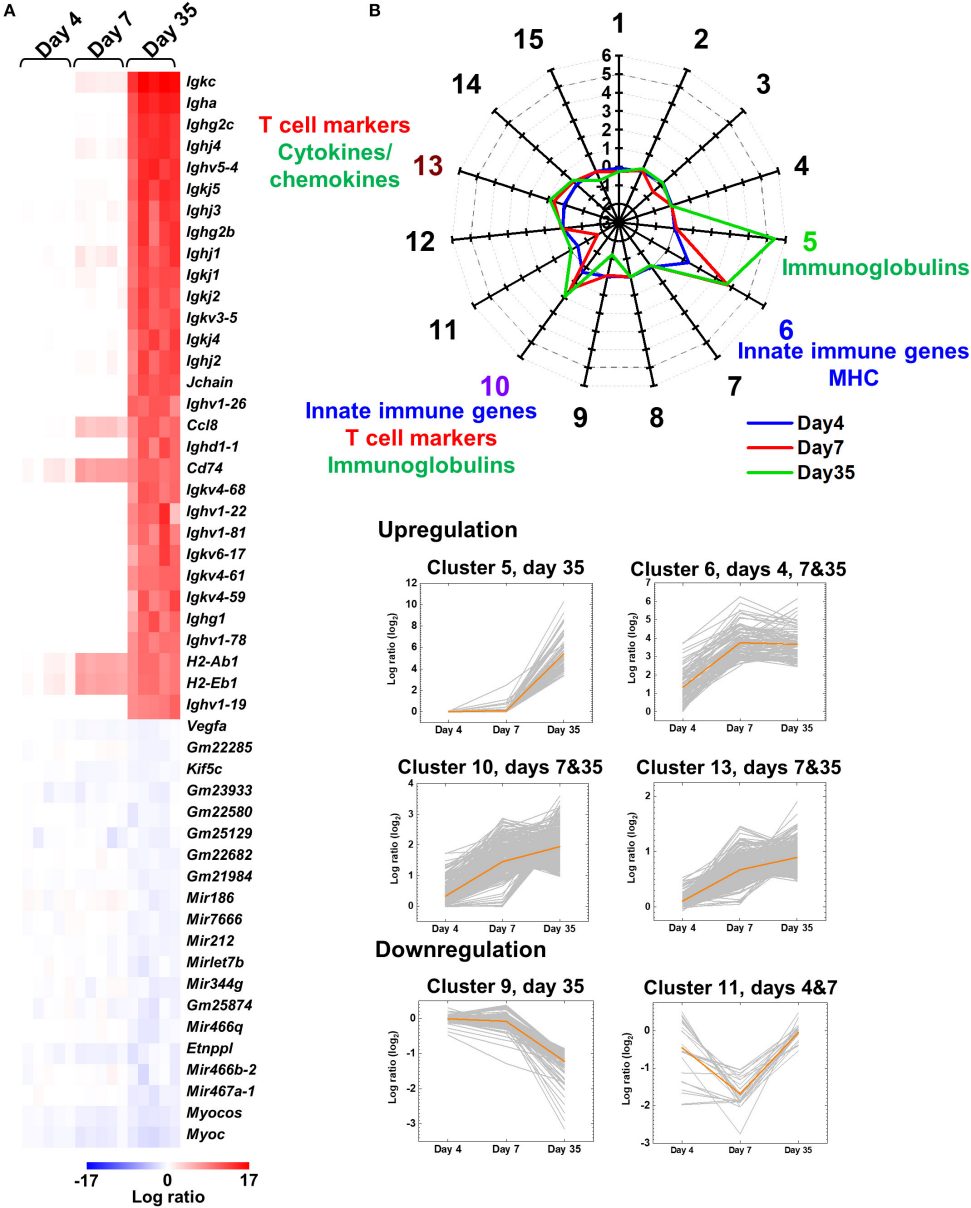
Central nervous system (CNS) transcriptome data of mice infected with Theiler's murine encephalomyelitis virus (TMEV). **(A)** We harvested the spinal cord from five TMEV-infected mice per day and analyzed the gene expression patterns compared with five control mice. We drew the heat map of the top 30 genes up- or the 20 genes downregulated in the spinal cord of TMEV-infected mice on day 35 compared with those on days 4 and 7 by the R packages “gplots” and “genefilter.” Red, blue, and white indicate upregulation, downregulation, and no change, respectively. Humoral immunity-related genes, including immunoglobulin (Ig) heavy (*Igh*), light [kappa (*Igk*) and lambda (*Igl*)], and J (*Jchain*) chains, were highly upregulated in TMEV-infected mice on day 35. Lists of significantly up and downregulated genes in TMEV-infected mice were shown in Supplemental Table 2 and Supplemental Figure 1. **(B)** Radar chart of cluster centers calculated by *k*-means clustering. *K*-means clustering, using an R package “cclust,” divided the genes into 15 clusters based on the expression patterns. Genes in cluster 5 were upregulated on day 35, which included Igs. Genes in cluster 6 were upregulated at all time points, which included innate immunity and major histocompatibility complex (MHC) genes. Genes in clusters 10 and 13 were upregulated on days 7 and 35; Cluster 10 included innate immunity genes, CD3 γ chain, and Igs. Cluster 13 included cytokines, chemokines, and T-cell-related genes. In contrast, genes in clusters 9 and 11 were downregulated on days 35 and 7, respectively. The numbers along the axis (−3 to 6) are log ratios, compared with controls. Lists of all genes classified into each cluster were shown in Supplemental Table 3.

A heat map was drawn to determine the expression patterns of top 30 upregulated and top 20 downregulated genes on day 35 (Figure 1) or at each time point (Supplemental Figure 1) in the spinal cord of TMEV-infected mice, and compared the expression levels with those on the other two time points, using R and the packages “gplots” and “genefilter.” We obtained the logarithmic fold-change data of each TMEV-infected mouse, compared with controls, using regulated-logarithm transformation (rlog) function, which added pseudocount “1” to all count data, in DESeq2 (37).

#### Principal Component Analysis (PCA)

PCA was conducted to compare the microbiome among the samples, using an R function “prcomp,” as we described previously (39). A graph of PCA with ellipses of an 80% confidence interval was drawn, using R packages “dplyr” and “ggplot2.” Factor loading for principal component (PC) 2 was used to rank a set of genes correlated with PC2 values.

#### Alpha Diversity

We determined alpha diversity of microbiome of fecal samples from TMEV-infected and control groups on days 4, 7, and 35 (*n* = 5 per group), using QIIME 2. To compare the number of genera, evenness, and the combination of them between groups, we used Faith's phylogenic diversity index, Pielou's evenness index, and Shannon index, respectively (40).

#### *K*-Means Clustering

*K*-means clustering was conducted to classify the genes based on their expression patterns, using an R package “cclust” (41). Davies-Bouldin index was used to determine the optimum number of clusters (Supplemental Figure 2) (42).

#### Pattern Matching

To evaluate the association between gut microbiota and CNS gene expressions, we conducted pattern matching by R (26) to identify correlations between spinal cord gene expressions (with statistical differences between TMEV-infected and control groups, *P* < 0.05) and relative abundance of the genus *Dorea* (day 4), the genus *Marvinbryantia* (days 7 and 35), or the genus *Coprococcus* (day 35) in fecal samples (*n* = 10 on days 4 and 7; *n* = 8 on day 35). We considered significantly strong positive or negative correlations when genes' correlation coefficients (*r*) were more than 0.8 or less than −0.8, respectively (calculated by R) with *P* < 0.05 (calculated by functions of Microsoft Excel, Microsoft Corporation, Redmond, WA).

### Enzyme-Linked Immunosorbent Assay (ELISA)

#### Anti-TMEV Antibody Isotype Determination

When the mice were killed, blood was collected from the heart of TMEV-infected mice. The levels of serum anti-TMEV isotype antibodies were assessed by ELISA as described previously (43). Ninety-six-well flat-bottom Nunc-Immuno plates, MaxiSorp surface (Thermo Fisher Scientific) were coated with TMEV antigen at 4°C overnight. After blocking with 10% fetal bovine serum (FBS) and 0.2% Tween 20 in PBS, 2^7^- or 2^11^-fold diluted serum samples were plated. Horseradish peroxidase (HRP)-conjugated anti-mouse IgG1 (Thermo Fisher Scientific), IgG2b (Thermo Fisher Scientific), IgG2c (SouthernBiotech, Birmingham, AL), or IgA (Thermo Fisher Scientific) was used to detect binding anti-TMEV isotype antibodies. The reaction was developed by adding *o*-phenylenediamine dihydrochloride (FUJIFILM Wako Pure Chemical Corporation, Osaka, Japan), and stopped with 1N HCl. Absorbance was read at 490 nm on a Model 680 Microplate Reader (Bio-Rad Laboratories, Hercules, CA).

#### Anti-*Coprococcus* Antibody

To investigate antibody responses to *Coprococcus*, we obtained *Coprococcus* sp. (RD014227) from the Culture Collection Division, Biological Resource Center, National Institute of Technology and Evaluation (NITE, Chiba, Japan) and cultured bacteria in modified Gifu Anaerobic Medium Broth (Nissui Pharmaceutical, Tokyo, Japan) with 1% glucose at 37°C for 3 days under an anaerobic condition, using the anaerobic cultivation set, AnaeroPack® (Mitsubishi Gas Chemical, Tokyo, Japan). We centrifuged cultured bacteria at 3,000*g* for 10 min, washed with PBS, and suspended the bacteria pellet in PBS [optical density (OD)_600nm_ = 8.0]. We sensitized mice with the bacterial solution (44) in PBS (200 μg bacterial protein/1 × 10^9^ bacterial cells/200 μL/mouse) intraperitoneally (i.p.) or with emulsified the bacterial solution in complete Freund's adjuvant (CFA) (final concentration: 200 μg bacterial protein/1 × 10^9^ bacterial cells/200 μL/mouse) subcutaneously, followed with a second bacterial i.p. injection 6 days after the first sensitization. Three days after the second challenge, we harvested the sera from the sensitized mice and used the sera as positive controls for anti-*Coproccocus* antibody ELISA. To prepare *Coprococcus* ELISA antigen, we centrifuged the bacterial solution, washed with PBS twice, and sonicated the pellet suspended in carbonate-bicarbonate buffer (pH 9.5) with 1% TritonX-100. Then, following centrifugation at 20,000*g* for 10 min, we collected the supernatant and used it as ELISA antigen. We coated Maxisorp F8 stripes (Thermo Fisher Scientific) with *Coprococcus* antigen (5 μg/well) overnight and added diluted mouse sera (1:10, 100 μL/well) from naive, TMEV-infected, or *Coprococcus*-sensitized mice. We detected anti-*Coprococcus* antibodies with HRP-conjugated goat anti-mouse IgG (H+L) (Thermo Fisher Scientific). We used the TMB substrate reagent set (BD Bioscience, Franklin Lakes, NJ) and measured absorbance at 450 nm.

#### Competitive ELISAs

Competitive ELISAs were performed as described previously (45, 46). Briefly, our standard anti-TMEV antibody ELISA was performed as described above with the following modifications: We incubated diluted sera from TMEV-infected mice with TMEV antigen, *Coprococcus* antigen, *Helicobacter pylori* antigen (47), or myelin oligodendrocyte glycoprotein (MOG)_35−55_ peptide (United BioSystems, Herndon, VA) (48) (final concentration of antigen, 20 μg/120 μL) in PBS containing 10% FBS and 0.2% Tween 20 at 4°C overnight. The mixtures were centrifuged at 10,000 rpm (7,707*g*) for 5 min, and the supernatants were reacted with TMEV antigen-coated wells.

### Histology

We perfused mice with PBS, followed by a phosphate-buffered 4% paraformaldehyde (FUJIFILM Wako Pure Chemical Corporation) solution, and harvested the spinal cord. We stained 4-μm paraffin sections with Luxol fast blue (Solvent blue 38; MP Biomedicals, Irvine, CA) for myelin visualization, as described previously (49). For IgA detection, we incubated the sections with rat anti-mouse IgA monoclonal antibody (1:200 dilution, clone 11-44-2, Beckman Coulter, Brea, CA) and anti-rat IgG-peroxidase (Nichirei Bioscience, Tokyo, Japan). We visualized the antibody/antigen complexes using 3,3'-diaminobenzidine (DAB, FUJIFILM Wako Pure Chemical Corporation). As negative and positive controls for spinal cord inflammatory demyelinating lesions, we used sections from naive mice or mice with experimental autoimmune encephalomyelitis (EAE) induced with myelin proteolipid protein (PLP) _139−151_ peptide in CFA as described previously (49).

### Statistics

Statistical analyses were conducted by calculating the Student *t*-test or analysis of variance (ANOVA), using the OriginPro 2018b (OriginLab Corporation, Northampton, MA). *P* < 0.05 was considered as a significant difference.

## Results

### Distinct Upregulation of TCR and Ig Genes During the Acute and Chronic Phases of TMEV Infection

TMEV has been known to induce a biphasic disease in the CNS: acute polioencephalomyelitis in the gray matter around 1 week p.i. and chronic inflammatory demyelinating disease in the white matter during the chronic phase, around 1 month p.i. Although the lesion distributions of the two phases differ (gray matter vs. white matter), it is unclear what factors could contribute to the difference. We determined the gene expression profiles in the CNS of TMEV-infected mice during the time course by RNA sequencing (24).

Prior to disease onset, on day 4, innate immunity-related genes, including chemokines and interferon (IFN)-induced genes, were upregulated in TMEV-infected mice (Supplemental Figure 1A and Supplemental Tables 1, 2A). During the acute phase, on day 7, in addition to continuous upregulation of innate immunity-related genes, T-cell-related genes, including TCRβ chain (*Trb*), were upregulated (Supplemental Figure 1B and Supplemental Table 2B). In contrast, during the chronic phase, on day 35, the heat map showed that most highly upregulated genes were humoral immunity-related genes, including Ig heavy (*Igh*) chains (IgA, IgG1, IgG2b, and IgG2c), light chains [kappa (*Igk*) and lambda (*Igl*)], and J (*Jchain*) chain (Figure 1A, Supplemental Figure 1C, and Supplemental Table 2C).

To classify the genes based on time-course expressions, we conducted *k*-means clustering (Figure 1B). *K*-means clustering of the CNS transcriptome data divided the genes into 15 clusters whose number was determined by Davies-Bouldin index (Supplemental Figures 2, 3). A radar chart of *k*-means clustering showed that genes in cluster 5 were upregulated on day 35, genes in cluster 6 were upregulated at all time points, and genes in clusters 10 and 13 were upregulated on days 7 and 35. Cluster 5 included a large number of Igs, such as *Igkc, Igha*, and *Ighg2c*. Cluster 6 included innate immune genes, such as IFN-induced proteins with tetratricopeptide repeats (*Ifit1* and *Ifit3*) and IFN regulatory factor 7 (*Irf7*), and MHCs, such as *H2-Q6, H2-K1*, and *H2-Ab1* (Supplemental Table 3). Cluster 10 included IFN-inducible genes, such as radical S-adenosyl methionine domain containing 2 (*Rsad2*), *Ifi44* and MX dynamin-like GTPases (*Mx1* and *Mx2*), CD3 γ chain (*Cd3g*), and Igs, such as *Igkj3, Igkv14-111*, and *Ighd4-1*. Cluster 13 included cytokines (e.g., *Il10, Il17ra*, and *Tnf* ), chemokines (e.g., *Ccl3, Ccl6*, and *Ccr1*), and T-cell-related genes (e.g., *Cd3d, Cd4*, and *Cd8a*). In contrast, genes in clusters 9 and 11 were downregulated in the TMEV-infected group on days 35 and 7, respectively. Representative genes in these clusters were as follows: cluster 9, mitochondrially encoded cytochrome c oxidases (*mt-Co1, 2*, and *3*), myelin proteolipid protein 1 (*Plp1*), and glycoprotein m6b (*Gpm6b*); cluster 11, microRNA (*Mir6236*), myelin protein zero (*Mpz*), and mitochondrial leucyl-tRNA synthetase (*Lars2*).

### No Changes in Overall Microbiome Patterns Among the TMEV-Infected and Control Groups

**Figure 2:**
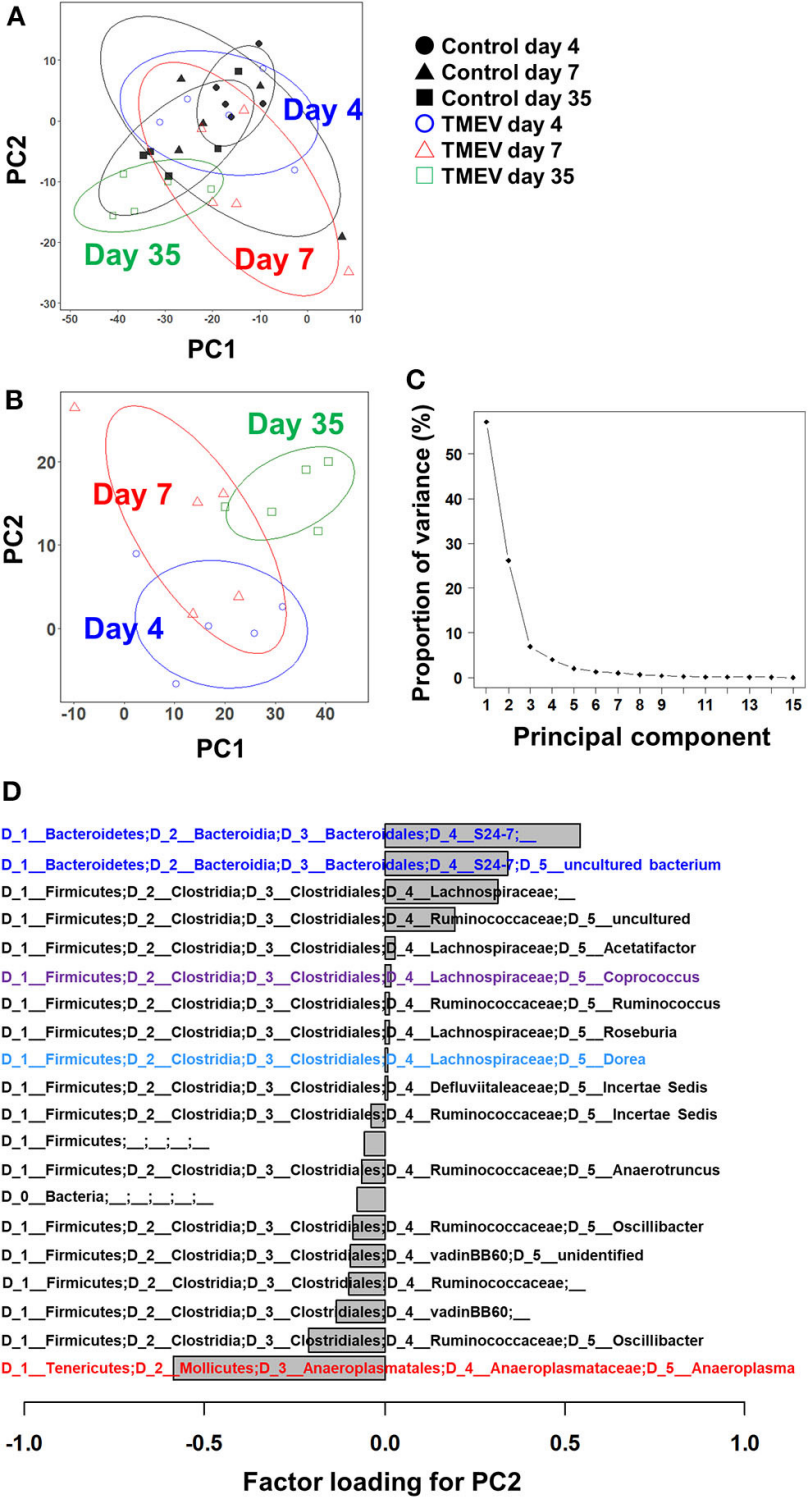
Principal component analysis (PCA) of fecal microbiome data from the TMEV-infected and control groups. **(A)** When PCA was conducted using all samples from TMEV-infected and control groups on days 4, 7, and 35, PCA did not separate any sample groups as a distinct population, although TMEV-infected group on day 35 had low principal component (PC) 1 and PC2 values. Ellipses indicated an 80% confidence interval of each group. **(B)** When PCA was conducted using only samples from TMEV-infected groups, PC2 values increased over the time course from day 4 to day 35. **(C)** Proportion of variance showed that PC1 and PC2 explained 56 and 25% of variance among the samples, respectively. **(D)** Factor loading showed the correlations between relative abundance of each bacterial taxon to PC2 values. Increased bacteria belong to the family *S24-7* and decreased bacteria belong to the genus *Anaeroplasma* correlated with PC2 values. We conducted PCA of fecal sample data (*n* = 5 per group at each time point), using an R function “prcomp.” Graphs with ellipses were drawn, using R packages “dplyr” and “ggplot2”.

To determine whether TMEV infection changes gut microbiota, we conducted 16S rRNA sequencing, using fecal samples. First, we compared overall microbiome patterns among the samples by conducting PCA of microbiome data from the TMEV-infected and control groups at all time points. Although all samples from the TMEV-infected group on day 35 had low PC1 and PC2 values, PCA did not separate any groups as a distinct population among the samples, suggesting no overall microbiota changes among the groups (Figure 2A). We also conducted PCA comparing the following sets of fecal microbiome data: (1) the control groups on days 4, 7, and 35 (Supplemental Figure 4A); (2) the TMEV-infected and control groups on day 4 (Supplemental Figure 4B); (3) the TMEV-infected and control groups on day 7 (Supplemental Figure 4C); and (4) the TMEV-infected and control groups on day 35 (Supplemental Figure 4D). We found that all control samples on days 4, 7, and 35 had similar PC1 and PC2 values and that TMEV samples were not separated as a distinct population at any time points by PCA.

Next, we conducted PCA using all microbiome data (days 4, 7, and 35) from the TMEV-infected groups. We found that PC2 values increased over the time course from day 4 to day 35 (Figure 2B) and that proportion of variance of PC1 and PC2 was 56% and 25% of variance among the samples, respectively (Figure 2C). Thus, PC2 values seemed to reflect the presence of inflammation in the CNS among the TMEV-infected groups. We calculated factor loading to rank which bacteria in microbiota could contribute to PC2 values (Figure 2D). At the genus level, we found that abundance of two taxa of bacteria that belong to the family *S24-7* correlated with PC2 values positively and that of the genus *Anaeroplasma* correlated with PC2 values negatively.

### No Decrease in Bacterial Diversity in TMEV Infection

**Figure 3:**
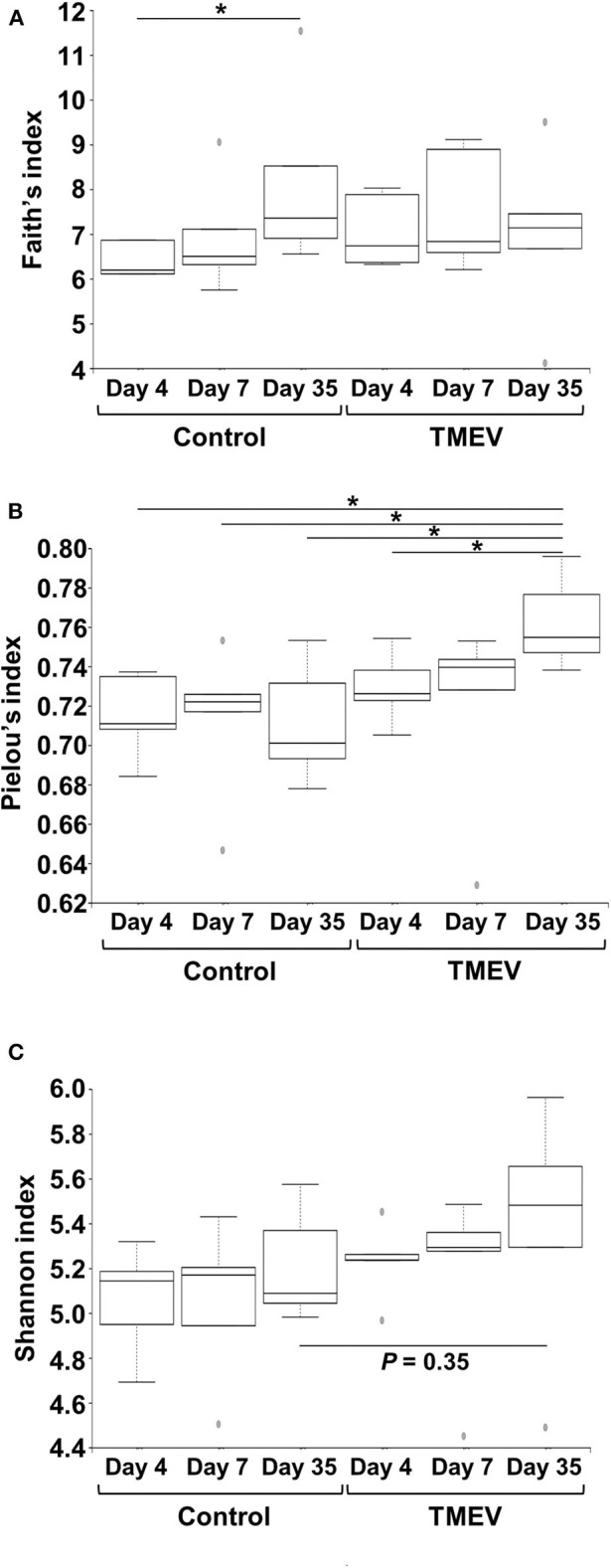
Time course analyses of bacterial alpha diversities of microbiome among TMEV-infected and control groups. Using QIIME 2, we compared the number of genera, evenness, and the combination of them between the groups (*n* = 5) by Faith's phylogenetic diversity index **(A)**, Pielou's evenness index **(B)**, and Shannon index **(C)**, respectively. **(A)** The numbers of bacterial genera differed significantly only between control groups days 4 and 35 (*P < 0.05, ANOVA). **(B)** We found significantly increased evenness in the TMEV-infected group on day 35 compared with all the other groups (*P < 0.05, ANOVA). **(C)** We found increased Shannon indices in TMEV-infected groups over the time course, but no statistical differences between the groups (*P* = 0.35, control vs. TMEV groups on day 35).

To compare the fecal microbial diversities among the TMEV-infected and control groups, we determined alpha diversity using three indices. In Faith's phylogenetic diversity index comparing the richness (the number of bacterial genera), we did not find significant differences between the TMEV-infected and control groups, but only between the control groups on days 4 vs. 35 (Figure 3A). In Pielou's evenness index comparing the evenness of amounts of bacterial genera, the index was increased significantly in the TMEV-infected group on day 35, compared with the other groups (*P* < 0.05, Figure 3B). In Shannon index comparing overall changes of diversity among the groups, we found increases in the indices in the TMEV-infected groups over the time course, but no statistical difference between the groups (*P* = 0.35, the TMEV-infected vs. control groups on day 35, Figure 3C).

### Three Bacterial Genera Changed in TMEV Infection

**Figure 4:**
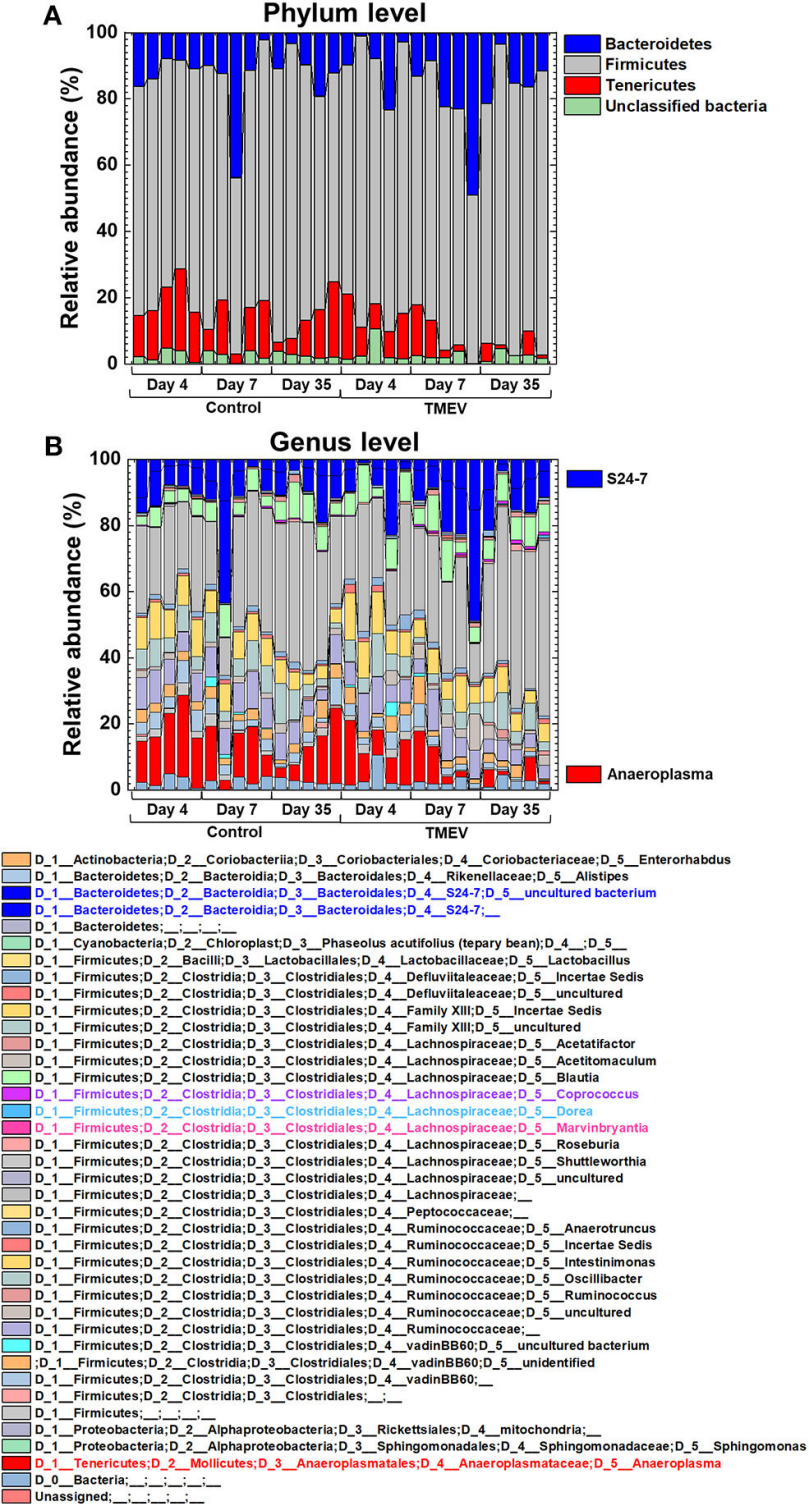
Relative abundance of fecal bacteria in the TMEV-infected and control groups. Fecal DNA was extracted and analyzed the microbiome by 16S rRNA sequencing. **(A)** At the phylum level, *Bacteroidetes* (blue) and *Tenericutes* (red) seemed to be increased and decreased in the TMEV-infected groups, respectively, without statistical differences. **(B)** At the genus level, on day 35, two taxa of the family *S24-7* (blue) and the genus *Anaeroplasma* (red) seemed to be increased and decreased in the TMEV-infected group, respectively, without statistical differences.

**Figure 5:**
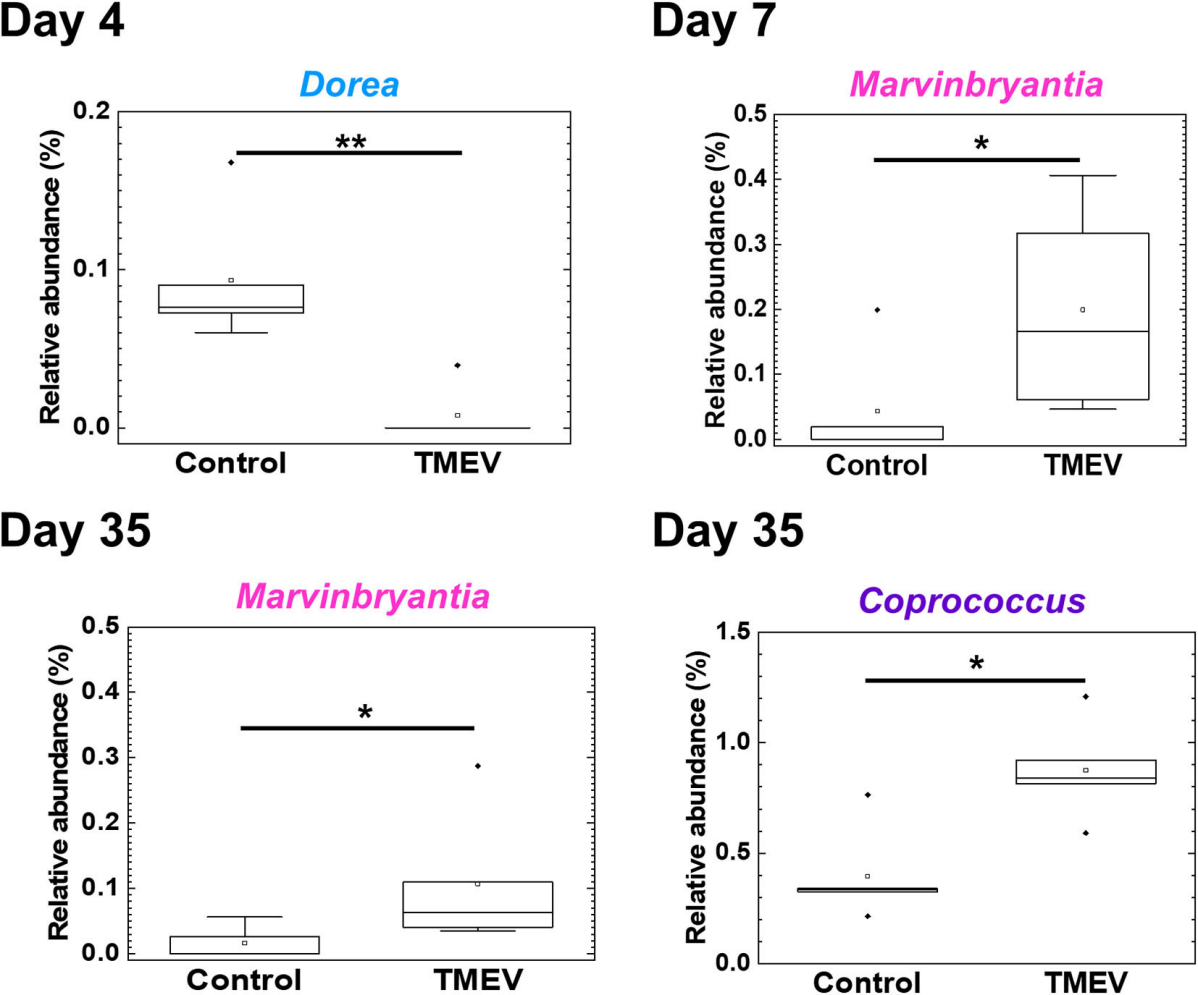
Three bacterial genera changed their relative abundance in the TMEV-infected group, compared with controls. Following the TMEV infection, the genus *Dorea* was decreased on day 4 (**P < 0.01) and the genus *Marvinbryantia* was increased on days 7 and 35 (*P < 0.05). The genus *Coprococcus* was increased on day 35 (*P < 0.05). We compared relative abundance of fecal microbes at the genus level using five fecal samples per group between TMEV-infected and control groups and determined the statistical differences using Student's *t*-test.

We drew a cumulative bar plot for relative abundance of microbiome in each sample. At the phylum level, although the phylum *Bacteroidetes* (Figure 4A, blue) and the phylum *Tenericutes* (Figure 4A, red) were upregulated and downregulated in TMEV-infected mice on day 35, respectively, the changes were not statistically significant. We also did not find significant changes in bacterial abundance at the order level in any groups (Supplemental Figure 5). At the genus level, although two taxa that belong to the family *S24-7*, phylum *Bacteroidetes* (Figure 4B, blue), and one genus *Anaeroplasma* (Figure 4B, red) that belongs to the phylum *Tenericutes* were increased and decreased in the TMEV-infected groups, respectively, the changes were not statistically significant. On the other hand, the TMEV-groups had significant differences in abundance of three distinct genera compared with the control group: a decrease in *Dorea* on day 4 (*P* < 0.01) (Figures 4B, 5, light blue); an increase in *Marvinbryantia* on days 7 and 35 (*P* < 0.05) (Figures 4B, 5, magenta); and an increase in *Coprococcus* on day 35 (*P* < 0.05) (Figures 4B, 5, purple).

### Correlations of Abundance of Three Bacterial Genera With Distinct Immune Gene Expressions in the CNS

**Figure 6:**
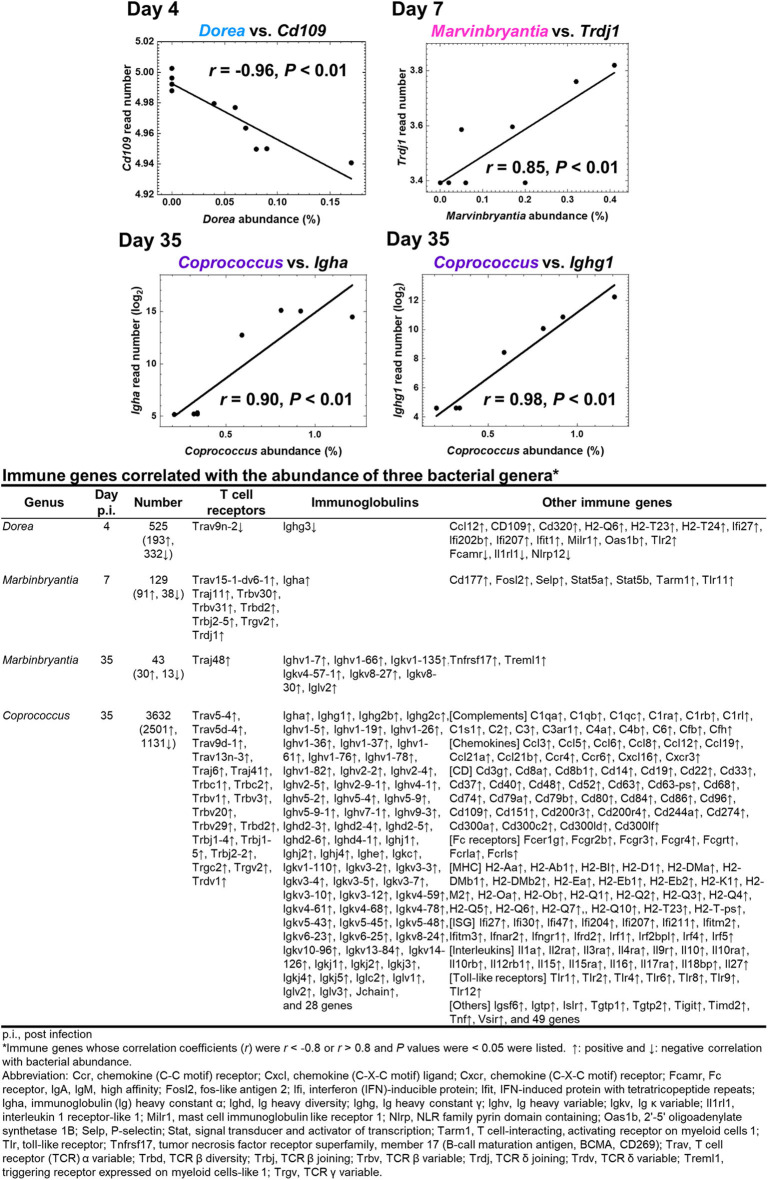
Correlations of relative abundance of three fecal bacterial genera with CNS gene expressions. The correlations were determined by pattern matching between fecal microbiome and CNS transcriptome data. (Top) Representative genes correlated with bacterial relative abundance: day 4, *Dorea*, negative correlation with *Cd109*; day 7, *Marvinbrynantia* positive correlation with TCRδ (*Trd*); and day 35, *Coprococcus* positive correlation with Ig heavy chain α gene (*Igha*) and Ig heavy chain γ1 (*Ighg1*). (Bottom) A list of representative genes correlated with relative abundance of three genera positively (↑, *r* > 0.8), or negatively (↓, *r* < −0.8); complete gene lists were shown in Supplemental Table 4. We calculated correlation coefficients between bacterial relative abundance vs. CNS gene expressions that were significantly different between control and TMEV groups by R and listed the correlated genes whose *P* values < 0.05.

To determine whether the changes in fecal microbiome were associated with gene expressions in the CNS, we conducted pattern matching between relative abundance of the three bacterial genera and CNS transcriptome data (Figure 6 and Supplemental Table 4). On day 4, abundance of the genus *Dore* correlated with 525 genes (193 upregulated and 332 downregulated genes), including CD109, in the CNS (Figure 6). On day 7, abundance of the genus *Marvinbryantia* correlated with 129 genes (91 upregulated and 38 downregulated genes); among immune genes, we found strong correlations with eight TCR genes, including TCR δ chain (*Trdj1*) and one IgA gene (*Igha*) (Figure 6). On the other hand, on day 35, abundance of the genus *Marvinbryantia* correlated with 43 genes (30 upregulated and 13 downregulated genes), which included only one TCR gene (*Traj48*) and seven genes of variable regions of Ig light or heavy chains. Additionally, on day 35, an abundance of the genus *Coprococcus* correlated with 3,632 genes (2,501 upregulated and 1,131 downregulated genes); we found strong correlations with 19 TCR genes and 93 Ig genes, which consisted of six constant region genes, including *Igha, Ighg1*, and *Ighg2c* and 87 variable regions of heavy and light chains (Figure 6). Most of these TCR and Ig genes were correlated with the genus *Coprococcus* only, although *Igha* and *Iglv2* expressions were also correlated with the genus *Marbinbryantia* on days 7 and 35, respectively. The abundance of the genus *Coprococcus* also correlated with other immune genes, particularly genes related to MHC class I and class II, complements, and toll-like receptors (TLRs) (Figure 6, list).

### Antibody Isotype Responses in TMEV Infection

The RNA-seq data of immune-related genes, including TCR and Ig, during the acute and chronic phases of TMEV infection were consistent with the findings previously reported using various methods, such as ELISA, flow cytometry, and immunohistochemistry in TMEV research (15, 50). We have validated representative RNA-seq data, using real-time PCR, including gene expressions of Ig heavy chains of IgG1, IgG2b, IgG2c, IgA, J chains, and TCRs (Supplemental Figure 6). Since TMEV-specific IgG-positive B and plasma cells have been demonstrated in TMEV infection (51), we anticipated upregulation of heavy chains (IgG1, G2b, and G2c) and κ light chain. On the other hand, the gene expression of IgA and J chain, reflecting the production of IgA dimers, components of mucosal immunity, was not anticipated in the CNS of TMEV infection.

**Figure 7:**
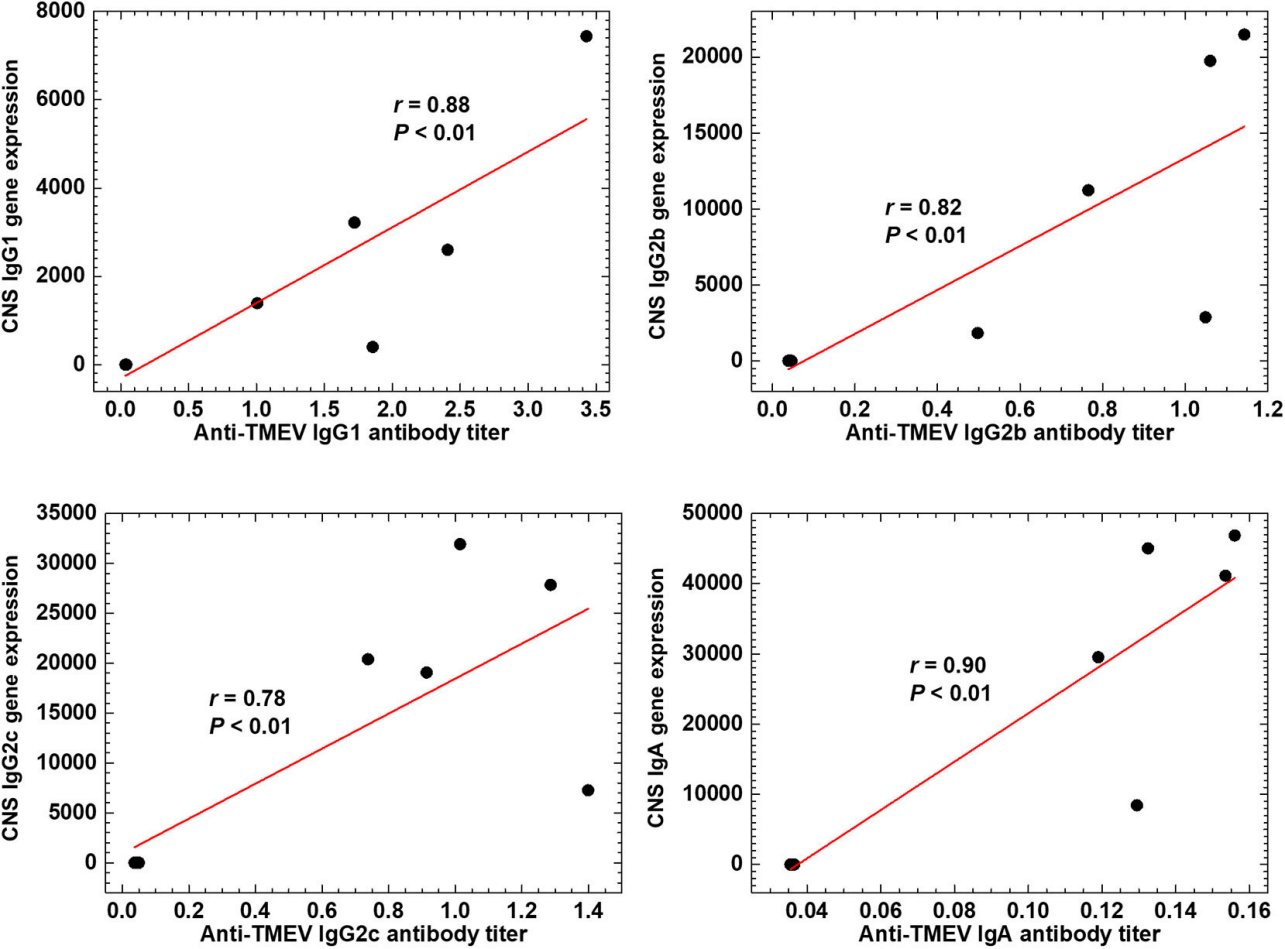
Anti-TMEV antibody isotype responses. Correlations between CNS Ig isotype expressions and serum anti-TMEV isotype antibody titers. During the chronic phase of TMEV infection, gene expression levels of constant regions of heavy chains of IgG1 (*Ighg1*), IgG2b (*Ighg2b*), IgG2c (*Ighg2c*), and IgA (*Igha*) were correlated with their anti-TMEV antibody isotype titers, significantly (*P* < 0.01, *n* = 10). We quantified the gene expression levels and anti-TMEV antibody titers by RNA-seq and enzyme-linked immunosorbent assays (ELISA, absorbance at 490 nm), respectively.

We tested whether the expression data of Ig genes could correlate with serum anti-TMEV IgG1, IgG2b, IgG2c, and IgA antibody titers (Figure 7). We found that all Ig isotype titers were correlated significantly with the gene expression data, suggesting that increased TMEV-specific Ig isotype responses explain the upregulation of Ig isotype gene expressions in the CNS. Thus, anti-TMEV antibody-producing cells seemed to infiltrate in the CNS, preferentially.

### IgA Deposition in the Spinal Cord During the Chronic Phase of TMEV Infection

**Figure 8:**
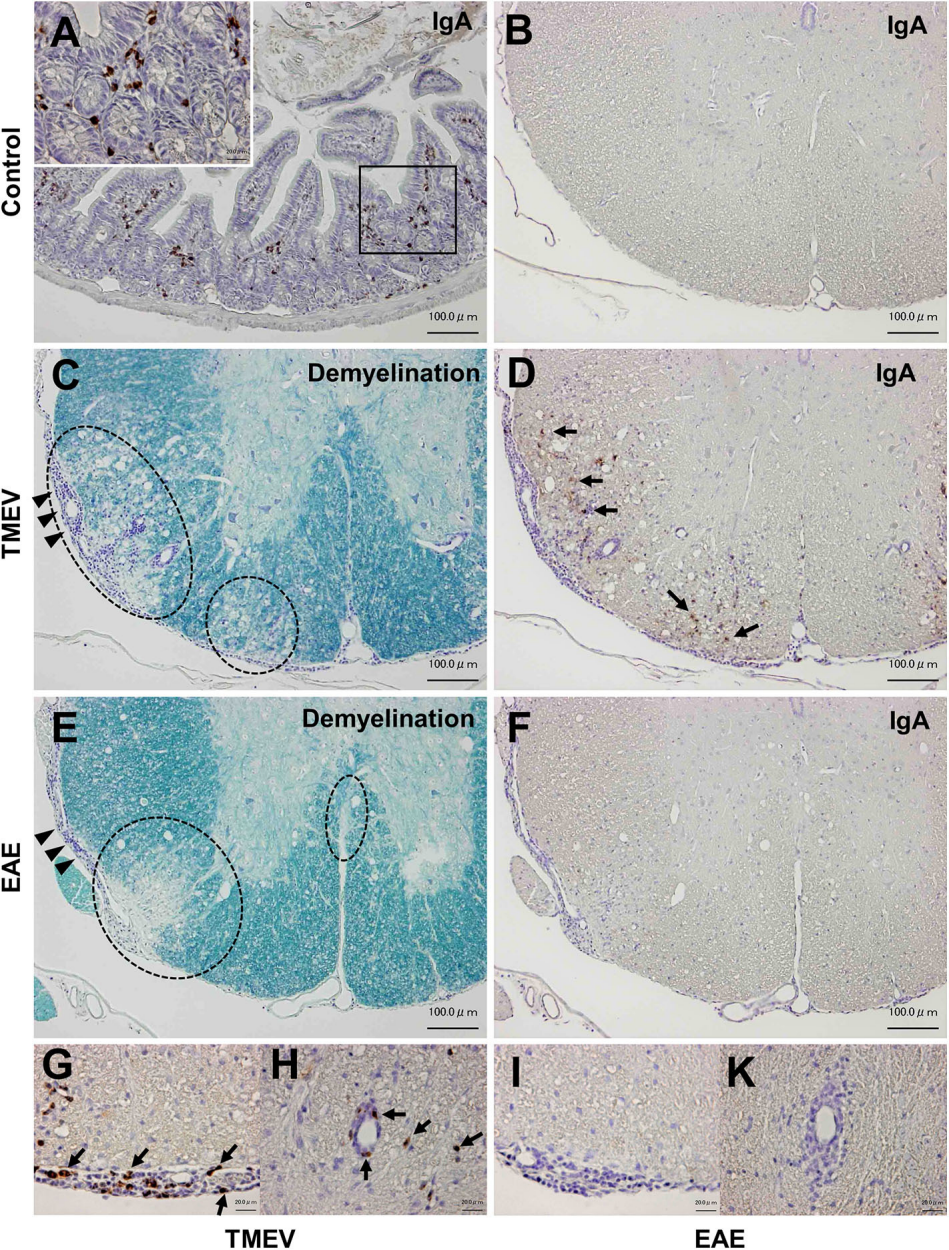
IgA immunohistochemistry in inflammatory demyelinating lesions in two models for multiple sclerosis. **(A)** We used IgA-producing cells in the lamina propria of the small intestine from uninfected control mice as positive controls for IgA immunohistochemistry. **(B)** In control mice, we did not detect IgA-positive cells in the spinal cord. **(C,D)** During the chronic phase of TMEV infection, inflammatory demyelinating lesions were present in the white matter of the spinal cord, where IgA strongly positive cells (arrows) were present in demyelinating parenchyma **(D)**, meningitis **(G)**, and perivascular cuffing **(H)**. IgA deposition was also observed in demyelinating lesions **(D)**. **(E,F)** Chronic inflammatory demyelinating lesions in the white matter of the spinal cord in experimental autoimmune encephalomyelitis (EAE) induced with myelin proteolipid protein (PLP). Despite the presence of inflammatory demyelination, there was no IgA immunoreactivity in the parenchymal **(F)**, meningeal **(I)**, or perivascular **(K)** inflammatory lesion of the spinal cord. Scale bar = 100 μm **(A–F)** and 20 μm **(G–K)**. **(A,B,D,F–K)**, IgA immunohistochemistry. **(C,E)**, Luxol fast blue stain. Dotted lines, demyelinating lesions. Arrowheads, meningitis. These sections are representative of naive, TMEV-infected, or EAE mice (*n* = 3–5 per group).

Although infiltration of T-cells and IgG-positive B cells in the CNS has been known during the acute and chronic phases of TMEV infection (15, 50), IgA responses have never been investigated in TMEV infection, since IgA response, in general, has been associated with mucosal immunity, not neuroinflammation. We investigated the localization of IgA-producing cells as well as IgA deposition using immunohistochemistry with sections of the intestine and spinal cord from uninfected control mice as positive and negative controls. In control mice, IgA-positive cells were present in the lamina propria of the intestine (Figure 8A), but absent in the spinal cord (Figure 8B). During the chronic phase of TMEV infection, we observed demyelinating lesions in the white matter of the spinal cord with perivascular cuffing (i.e., inflammation) and meningitis (Figure 8C). In the demyelinating lesions, we detected a substantial number of intense IgA-positive small round cells and larger cells with abundant cytoplasm, which were present in the meninges and perivascular cuffing, and infiltrating in the parenchyma (Figures 8D,G,H). We also detected extracellular IgA deposition in the demyelinating lesions. During the acute phase, TMEV induced inflammation in the gray matter and meninges, where IgA staining was not evident (data not shown). We also tested whether IgA positive cells could be seen in chronic inflammatory demyelinating lesions of the autoimmune model for MS, EAE induced with PLP sensitization in SJL/J mice. Although we observed inflammatory demyelinating lesions in the spinal cord of EAE mice, comparable to TMEV-induced demyelination (Figure 8E), IgA staining was undetectable in the EAE lesions (Figures 8F,I,K).

### No Cross-Reactive Antibody Isotype Responses to *Coprococcus* in TMEV Infection

**Figure 9:**
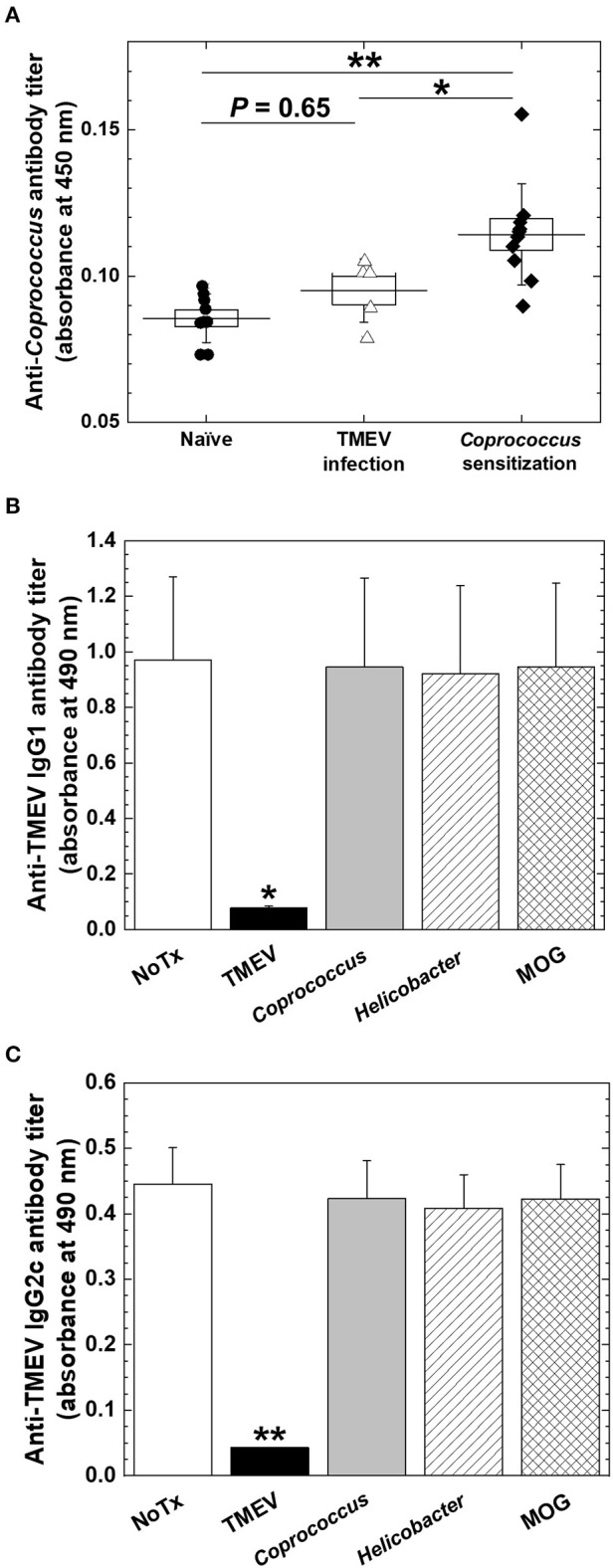
Anti-*Coprococcus* antibody responses in TMEV infection. **(A)** Using ELISA plates coated with *Coprococcus* antigen, we titrated serum anti-*Coprococcus* antibody responses in naive mice (*n* = 7), chronically TMEV-infected mice (*n* = 5), and *Coprococus*-sensitized mice (*n* = 10). We did not find significantly increased anti-*Coprococcus* antibody responses in TMEV infection (*P* = 0.65, ANOVA, compared with naive mice). *P < 0.05, **P < 0.01, ANOVA, compared with *Coprococcus*-sensitized mice. **(B,C)** We conducted anti-TMEV IgG1 **(B)** and IgG2c **(C)** isotype antibody ELISAs using sera in chronic TMEV infection. We incubated the sera overnight in the absence (None, white bar) or the presence of TMEV antigen (black bar), *Coprococcus* antigen (gray bar), *Helicobacter pylori* antigen (hatched bar), or myelin oligodendrocyte glycoprotein (MOG) peptide (cross-hatched bar). Only the adsorption of TMEV antigen decreased anti-TMEV antibody isotype responses. Shown are mean + SE of antibody titers of five sera per group.

*Coprococcus*, a component of human fecal microbiota, has been shown to potentially associate with humoral immune responses. Antibody responses to *Coprococcus* have been reported to differ in patients with IBD, compared with controls. *Coprococcus* has also been reported to interact with immunoglobulin *in vitro*. Since the gene expressions of several Ig heavy chain isotypes were correlated with *Coprococcus* abundance during the chronic phase of TMEV infection, we investigated how *Coprococcus* could affect antibody responses in TMEV infection. First, we established the ELISA system to detect anti-*Coprococcus* antibody, using sera from *Coprococcus*-sensitized mice as positive controls (44). We found that, although some sera from TMEV-infected mice seemed to react *Coprococcus* antigens weakly, we did not observe significant anti-*Coprococcus* antibody responses in sera from TMEV-infected mice (Figure 9A). Next, we tested whether anti-TMEV antibodies could cross-react with *Coprococcus* antigen. We incubated sera from TMEV-infected mice with *Coprococcus* antigen, positive control TMEV antigen, or two negative control antigens: *H. pylori* antigen or MOG peptide. Following overnight incubation, we conducted anti-TMEV isotype antibody ELISA to see whether adsorption with these antigens could decrease serum TMEV isotype antibody responses. Only the adsorption of TMEV antigen decreased anti-TMEV antibody isotype responses (IgG1, Figure 9B; IgG2c, Figure 9C; and IgA, data not shown). Thus, anti-TMEV antibodies did not cross-react with *Coprococcus* antigen *in vitro*.

## Discussion

In the current study, we first demonstrated distinct gene expression patterns on days 4, 7, and 35, using TMEV-infected CNS transcriptome data by *k*-means clustering with a radar chart (Figure 1). We found that T-cell-related genes, including TCR genes during the acute phase and Ig-related genes during the chronic phase, were highly upregulated, comprising distinct clusters 13 and 5, respectively. Next, we investigated that changes in overall gut microbiome (i.e., dysbiosis) could be associated with these unique gene expression patterns during the time course of TMEV infection, using PCA and alpha diversity indices.

Although overall changes in microbiome patterns by PCA have been demonstrated in many diseases having associations of microbiota with pathophysiology, we did not identify samples from TMEV-infected groups as distinct population by PCA (Figure 2). We found increased PC2 values over the time course by PCA of only TMEV microbiome data; the PC2 values might reflect the presence of CNS inflammation. The PC2 values were correlated with increased two taxa of the family *S24-7* and decreased *Anaeroplasma* genus. The family *S24-7*, which belongs to the phylum *Bacteroidetes*, is anaerobic, non-motile, Gram-negative bacteria and present within the human gut (52). The genus *Anaeroplasma*, which belongs to the phylum *Tenericutes*, is anaerobic, non-motile, and Gram-negative bacteria (53). The analysis of relative abundance of bacteria at the genus level also showed increased *S24-7* and decreased *Anaeroplasma* genera over the course of TMEV infection (Figure 2B). However, since there were no statistical differences in relative abundance of these bacteria among TMEV and control groups, the pathogenic roles of *S24-7* and *Anaeroplasma* could be limited, although they might play a minor role in CNS inflammation following TMEV infection.

We observed no significant changes in alpha diversity of microbiome by Shannon index in TMEV-infected mice, although we found increases in evenness by Pielou's index over the time course (Figure 3). In most microbiota-associated diseases and health conditions, such as IBD and obesity, alpha diversity has been reported to be decreased (54, 55). In TMEV infection, one research group also reported the decreased diversity in fecal microbiota (56, 57). Although the difference between our results and others might be due to the environment and location of mouse breeding such as diet and stress (58), our results were consistent with findings in human MS by Miyake et al. (59), in which the diversity did not show significant difference in the feces of MS patients. In human AFM, two research groups examined microbial flora, using nasopharyngeal swabs or CSF samples to find the pathogens to cause AFM, rather than to characterize microbiome (60, 61), where no consistent changes in microbiota were observed because of a limited number and amount of samples. Breitwieser et al. (60) identified potential involvement of *Haemophilus influenzae* and *Staphylococcus aureus* in one swab sample each and discussed that bacterial infections or their immune responses may trigger pathogenic processes.

Since overall changes in gut microbiome seemed not to contribute to TMEV pathophysiology, we tested whether relative abundance of distinct bacterial genera could be associated with TMEV-induced neuroinflammation. During the time course of TMEV infection, we identified the significant changes of abundance of three bacterial genera, *Dorea, Marvinbryantia*, and *Coprococcus* in TMEV-infected group (Figure 5). All the three genera, which were isolated from human feces, are anaerobic, non-motile and non-spore-forming Gram-positive bacteria and belong to the family *Lachnospiraceae* (62–65). Using pattern matching (Figure 6), we found strong correlations between relative abundance of the three bacterial genera and CNS gene expressions, including immune-related genes. On day 4, decreased *Dorea* correlated with expressions of several immune-related genes, including CD109 (66). Abundance of the genus *Marvinbryantia* correlated with eight TCR genes, including α, β, γ, and δ chains, only one Ig gene on day 7, and with only one TCR gene and seven Ig genes on day 35. These results were consistent with the heat map and *k*-means clustering data, where TCR and Ig genes were most highly upregulated in the CNS on days 7 and 35, respectively. Except for *Igha*, all TCR and Ig genes correlated with *Marvinbryantia* abundance were variable regions of TCR and Ig genes. It will be interesting to test whether these TCR and Ig genes could be used to recognize *Marvinbryantia* and, if so, whether the TCRs and Igs could also recognize TMEV antigens by molecular mimicry between bacterial and viral antigens (67).

Although the genus *Marvinbryantia* correlated mainly with a limited number of TCR and Ig genes, abundance of the genus *Coprococcus* correlated strongly with a larger number of TCR and Ig genes: 19 TCR genes and 93 Ig genes including IgA and J chains. Most of the TCR and Ig genes were correlated with the genus *Coprococcus* only, although *Igha* and *Iglv2* expressions were also correlated with the genus *Marbinbryantia* on days 7 and 35, respectively. *Marbinbryantia* abundance was related to mostly variable regions of a small number of TCR or Ig genes. On the other hand, *Coprococcus* abundance correlated with both variable and constant regions of Ig; increased *Coprococcus* may be associated with polyclonal activation of B cells. In addition, we found significant associations of *Coprococcus* abundance with other immune genes, including MHC class I and II and complements as well as chemokines, cytokines, and TLRs (Figure 6, bottom list). Thus, increased *Coprococcus* may be linked to a more general pro-inflammatory condition involving not only T and B cells, but also other immune components including antigen presenting cells.

The upregulated immune genes in the CNS were likely TMEV-specific, since upregulation of TMEV-specific T-cell and antibody responses have been demonstrated in the CNS during the acute and chronic phases of TMEV infection (15, 50). Consistent with previous findings, we found that the upregulated IgG-isotype gene expressions were highly correlated with TMEV-specific IgG isotype titers (Figure 7). For the first time, we demonstrated that increased IgA gene expression was correlated with anti-TMEV IgA titer. Using immunohistochemistry, we found that IgA-positive cells and IgA deposition were observed in and around inflammatory demyelinating lesions (Figure 8). Currently, we do not know whether IgA in the lesions plays a protective or pathogenic role in the CNS of TMEV infection. In EAE, an autoimmune model for MS, recirculating intestinal IgA-producing cells have been demonstrated to regulate neuroinflammation (68).

Although we do not know how certain bacteria in the gut can influence the gene expressions in the CNS, one straightforward explanation is that gut bacteria affect mucosal immunity, resulting in the activation of systemic immune cells, which leads to CNS infiltration. Among the three bacteria with altered abundance in the gut microbiota in TMEV infection, the physiological and pathogenic roles of *Dorea* and *Marvinbryantia* genera are largely unknown. On the other hand, the genus *Coprococcus* is a component of human fecal microbiota (69, 70), and their increased abundance was observed in patients with neuromyelitis optica (NMO), but not in MS, compared with healthy controls (71, 72). NMO is an inflammatory demyelinating disease, in which autoantibody against the water channel protein aquoaporin-4 has been associated with pathogenesis. In NMO, other bacteria including *Clostridium perfringens* were also significantly increased in feces, where several immunoregulatory roles of the bacteria have been proposed, although the role of the genus *Coprococcus* is unknown.

To clarify the role of *Coprococcus* in TMEV infection, we were not able to use standard approaches using germ-free (GF) mice or antibiotic cocktail treatment (73–77), since (1) GF mice and antibody treatment have been shown to increase blood–brain barrier (BBB) permeability (78); (2) GF mice have several immunological abnormalities even in naive mice, for example, TLR responses and T helper (Th) 17 responses which have been shown to play a key role in TMEV infection (43, 79); (3) depletion of microbiota by antibiotic cocktail have been shown to be lethal in TMEV infection (56); and (4) mono-colonization of GF mice with the non-spore-forming anaerobic *Coprococcous* is technically difficult. Thus, we decided to take a different approach by investigating antibody responses to *Coprococcus*.

Previously, agglutinating antibodies to *Coprococcus* have been found more frequently in sera of patients with Crohn's disease (44, 80, 81). Sera from Crohn's disease had not only specific *Coprococcus* binding of IgG through Fab portion, but also non-specific *Coprococcus* binding through Fc portions, the latter of which was similar to *Staphylococcus aureus* protein A (80). Thus, *Coprococcus* potentially has unique interactions with antibodies. Here, we tested whether (1) anti-TMEV isotype antibodies could cross-react with *Coprococcus* and (2) sera from TMEV-infected mice contained anti-*Coprococcus* antibody titers. In the current experiments, however, we found neither cross-reactivities between anti-TMEV vs. anti-*Coprococcus* antibodies nor induction of anti-*Coprococcus* antibody responses in TMEV infection, although these negative results could be attributed to technical difficulties in extraction of *Coproccoccus* antigen (44). Thus, we do not know how increased *Coprococcus* could be associated with antibody responses. *Coprococcus* might have a function to increase antibody isotype responses, particularly IgA production, which have been reported in other gut bacteria including *Clostridia* and segmented filamentous bacteria, previously (76). In EAE, a two-phase scenario of neuroinflammation has been proposed, where (1) gut microbiota initiates/expands autoimmune Th17 responses in gut-associated lymphatic tissue (GALT), then, (2) this leads to Th17-cell infiltration into the CNS (73). Similarly, in TMEV infection, distinct gut microbiota might initiate/expand IgA-producing cells in GALT, leading to IgA-producing cell infiltration and IgA deposition in the CNS.

Other than alterations of immune responses by microbiota, there are several possibilities that microbiota can affect viral diseases. For example, bacterial components, including lipopolysaccharide, have been shown to promote enteric virus replication and systemic pathogenesis (82). In addition, different enzymes from various bacteria have been shown to enhance virus infectivity and pathogenicity *in vivo* (83, 84). We performed predictive metagenome profiling, using microbiome data of TMEV-infected groups by PICRUSt. We were able to predict the changes in two pathways, arachidonic acid metabolism and ether lipid metabolism; we identified sets of bacteria that belong to the order *Clostridiales* were involved in the two pathways (Supplemental Figure 7). Currently, however, we do not know how these pathway changes in the gut microbiota could affect neuroinflammation in TMEV infection, although it is intriguing that changes in arachidonic and lipid metabolism in the CNS have been reported in MS ant the TMEV model (85–89).

**Figure 10:**
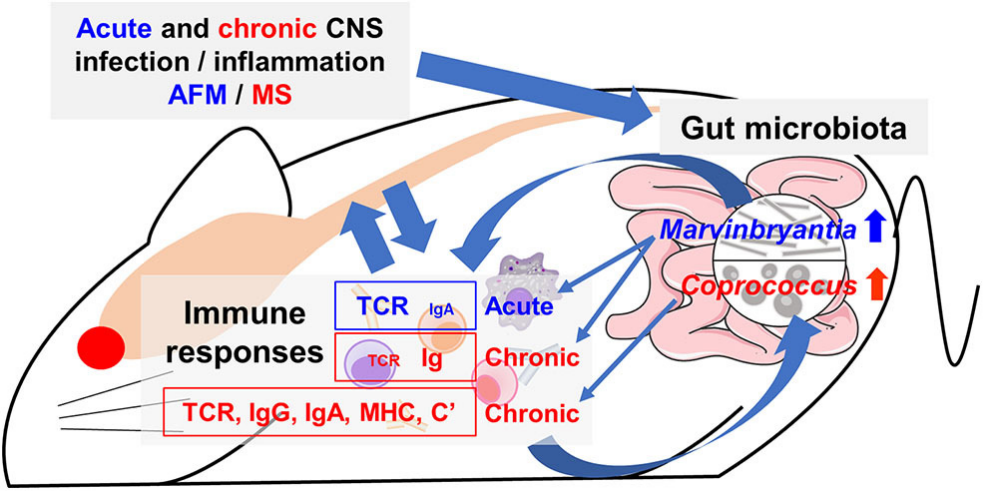
Working hypothesis of communication between the CNS and gut microbiota mediated by immune system. Acute or chronic CNS infection TMEV results in inflammation of the gray matter or white matter of the spinal cord, leading to diseases mimic acute flaccid myelitis (AFM) or multiple sclerosis (MS), respectively. Alteration of a limited number of distinct bacteria in gut microbiota was associated with different immune gene expressions during the acute and chronic phases. The genus *Marvinbryantia* abundance correlated with gene expressions of distinct T-cell receptors (TCR) and immunoglobulin (Ig) A during the acute phase and those of Igs during the chronic phase. The genus *Coprococcus* abundance was associated not only TCR and Ig (IgG isotypes and IgA) but also other immune related genes, including major histocompatibility complex (MHC) and complements (C'). Here, alteration of a small number of gut bacteria may affect distinct immune responses and then lesions in the CNS, although we do not know the precise pathophysiology, particularly the CNS upregulation of IgA, which is a component of mucosal immunity. Alternatively, activated immune response would alter composition of gut microbiota.

In conclusion, we found changes of a limited number of distinct bacterial genera in feces during the acute and chronic phases of TMEV infection. Each bacterial genus identified was associated with upregulation of different TCR and IgG/IgA gene expressions in the spinal cord (Figure 10). This is the first report suggesting that a limited number of gut bacteria could be associated with distinct TCR and Ig gene expressions and influence the inflammatory events, particularly IgA upregulation, which were observed in AFM-like and MS-like diseases in the spinal cord. However, we cannot rule out an alternative scenario for our findings in which activated immune responses could be altering the composition of the gut microbiota. Future experiments will further tease out these time-dependent communications between certain gut bacteria and the immune system, providing possible therapeutic strategies for the management and treatment of inflammation associated with AFM and MS-like diseases.

## Data Availability Statement

The datasets generated for this study can be found in the Gene Expression Omnibus (GEO) at the National Center for Biotechnology Information (NCBI, Bethesda, MD, USA; accession no. GSE120041, https://www.ncbi.nlm.nih.gov/geo/query/acc.cgiacc=GSE120041) and the Sequence Read Archive (SRA) at NCBI (Bioproject accession no. PRJNA561088; Biosample accession no. SAMN12607114-SAMN12607143).

## Ethics Statement

All animal experiments were approved by the Louisiana State University Health Sciences Center-Shreveport (LSUHSC-S, LA, USA) and the Kindai University Faculty of Medicine (Osaka, Japan) Institutional Animal Care and Use Committee (IACUC) guidelines and followed the National Research Council's Guide for the Care and Use of Laboratory Animals, the Institute of Laboratory Animal Resources (ILAR), and the guideline ‘Fundamental Guidelines for Proper Conduct of Animal Experiment and Related Activities in Academic Research Institutions' from the Ministry of Education, Culture, Sports, Science and Technology, Japan.

## Author Contributions

IT, KN, and FG conceived and supervised the project. FS, SK, YN, AK, and IT designed and conducted immunological experiments. SO and MF conducted bioinformatics analyses. A-MP conducted immunohistochemistry and *Coprococcus* experiments. SO and IT wrote the manuscript. All authors read and approved the final manuscript.

## Conflict of Interest

The authors declare that the research was conducted in the absence of any commercial or financial relationships that could be construed as a potential conflict of interest.
