# Supplementary material for: Bioinformatics Analysis of Gut Microbiota and CNS Transcriptome in Virus-Induced Acute Myelitis and Chronic Inflammatory Demyelination; Potential Association of Distinct Bacteria With CNS IgA Upregulation
Source: Front Immunol. 2020 Jul 7;11:1138. doi: 10.3389/fimmu.2020.01138 (PMC7358278; doi:10.3389/fimmu.2020.01138)
Supplement: Supplementary file 1 [file Data_Sheet_1.zip › Supplemental Material.docx]

**Supplemental Material**

**Table of contents**

**Supplemental Figure 1.** Heat maps of most highly up- or down-regulated genes in the spinal cord of TMEV-infected group …2

**Supplemental Figure 2.** Davies-Bouldin index to determine the number of clusters for *k*-means clustering …3

**Supplemental Figure 3.** The gene expression patterns of 15 clusters determined by *k*-means clustering of CNS transcriptome data …4

**Supplemental Figure 4.** Principal component analysis (PCA) of fecal microbiome data from TMEV-infected and control groups …5

**Supplemental Figure 5.** Relative abundance of bacteria in the feces from TMEV-infected and control groups at the order level …6

**Supplemental Figure 6.** Validation of RNA-seq data using real-time PCR …7

**Supplemental Figure 7.** Predictive metagenome profiling using microbiome data of TMEV-infected groups by PICRUSt. …8

**Supplemental Table 1.**  Functional clustering of CNS transcriptome data using DAVID …9

**Supplemental Table 2.** Lists of up- or downregulated genes in TMEV-infected mice on days 4, 7, and 35 …Supplemental Table 2.xlsx

**Supplemental Table 3.** Lists of genes in each cluster of *k*-means clustering

…Supplemental Table 3.xlsx

**Supplemental Table 4.** Lists of CNS genes pattern-matching with three bacterial genera

…Supplemental Table 4.xlsx

**Supplemental Table 5.** Sequences of primer sets for real-time PCR …10

**Supplemental Methods**  Predictive metagenome analysis …11

**Supplemental References** …14


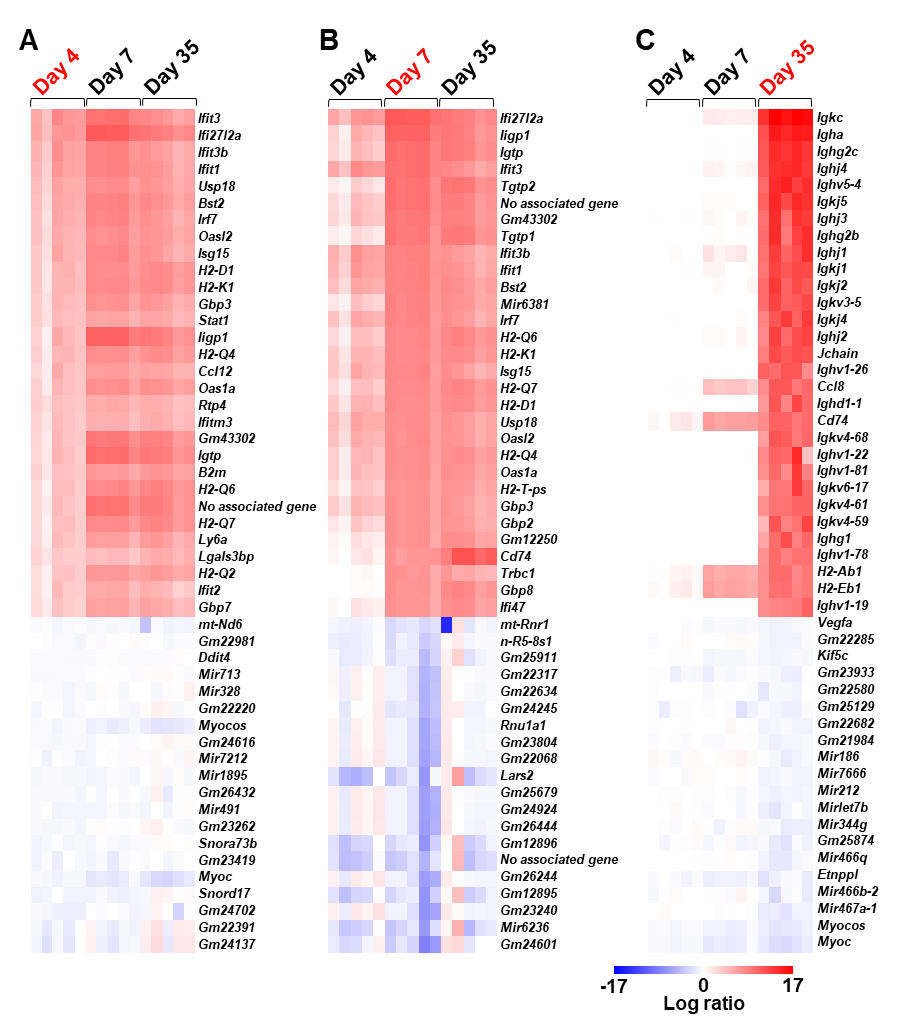


**Supplemental Figure 1.** **Heat maps of most highly up- or down-regulated genes in the spinal cord of TMEV infection.**

We infected SJL/J mice with Theiler’s murine encephalomyelitis virus (TMEV) and harvested the spinal cord on days 4 (A), 7 (B) and 35 (C) post infection (p.i.). A) We drew the heat map of top 30 genes up- or 20 genes down-regulated in the spinal cord of TMEV-infected mice on day 4 compared with those on days 7 and 35 by the R packages ‘gplots’ and ‘genefilter’. Innate immunity-related genes were highly upregulated at all time point. B) When we compared the top 30 genes up- or 20 genes down-regulated genes on day 7 with those on days 4 and 35, major histocompatibility complex (MHC) molecules and T cell-related genes were highly upregulated on days 7 and 35. C) When we compared the top 30 genes up- or 20 genes down-regulated genes on day 35 with those on days 4 and 7, immunoglobulins were highly upregulated only on day 35. Red, blue, and white indicate upregulation, downregulation, and no change, compared with control groups, respectively. Each column represents the data from one mouse. *n* = 5 per time point.

**Supplemental Figure 2. Davies-Bouldin index to determine the number of clusters for *k*-means clustering.**

We calculated Davies-Bouldin index and decided to use 15 clusters because the index showed the lowest score for 15 clusters.


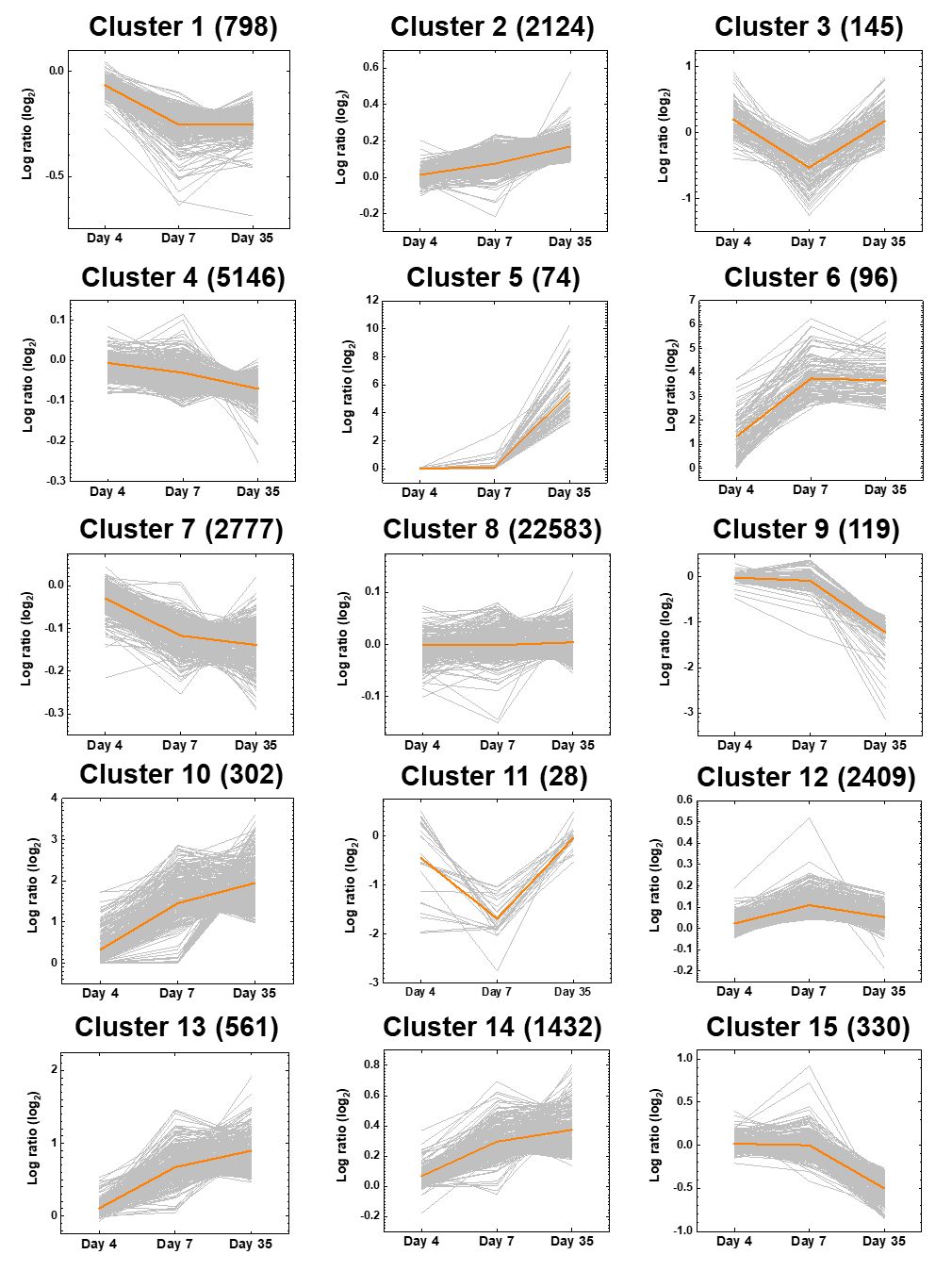


**Supplemental Figure 3. Gene expression patterns of 15 clusters determined by *k*-means clustering of transcriptome data.**

Graphs were drawn using all genes or 300 genes (when the total number of genes exceeds 300) in each cluster. The number next to a cluster number indicates the number of genes in each cluster. Orange line indicates cluster center in each cluster.


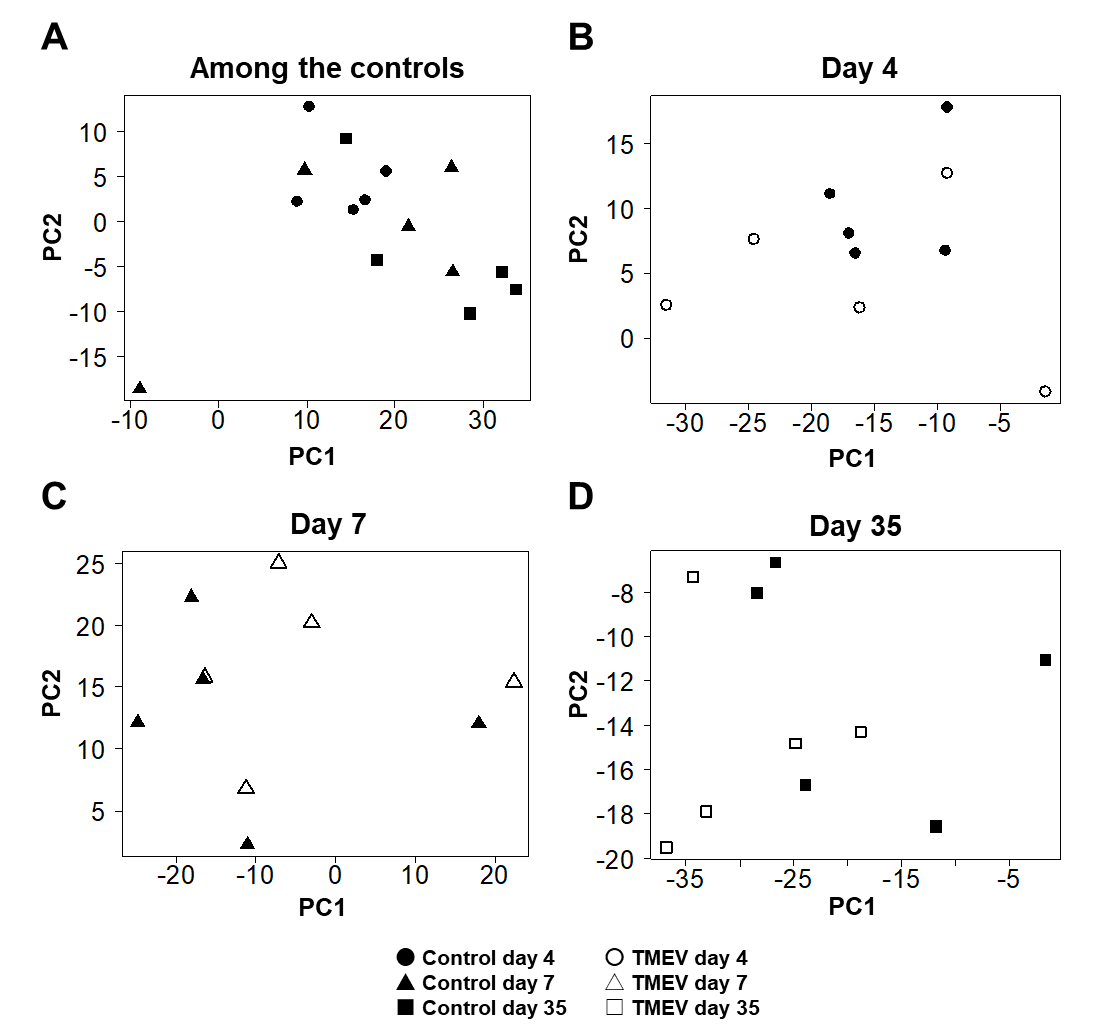


**Supplemental Figure 4.** **Principal component analysis (PCA) of fecal microbiome data from TMEV-infected and control groups.**

(A) PCA of all fecal microbiome data from control groups on days 4, 7, and 35. All control samples on days 4, 7, and 35 had similar principal component (PC)1 and PC2 values. (B) PCA of TMEV-infected and control groups on day 4. (C) PCA of TMEV-infected and control groups on day 7. (D) PCA of TMEV-infected and control groups on day 35. PCA did not separate TMEV samples as a distinct population at any time points.

**Control**

**TMEV**

**Day 35**

**Day 7**

**Day 4**

**Day 35**

**Day 7**

**Day 4**

**Supplemental Figure 5.** **Relative abundance of bacteria in the feces from TMEV-infected and control groups at the order level.**

Although no bacterial order showed significant differences, *Bacteroidales* (blue) were increased and *Anaeroplasmatales* (red) were decreased in TMEV-infected group.

**
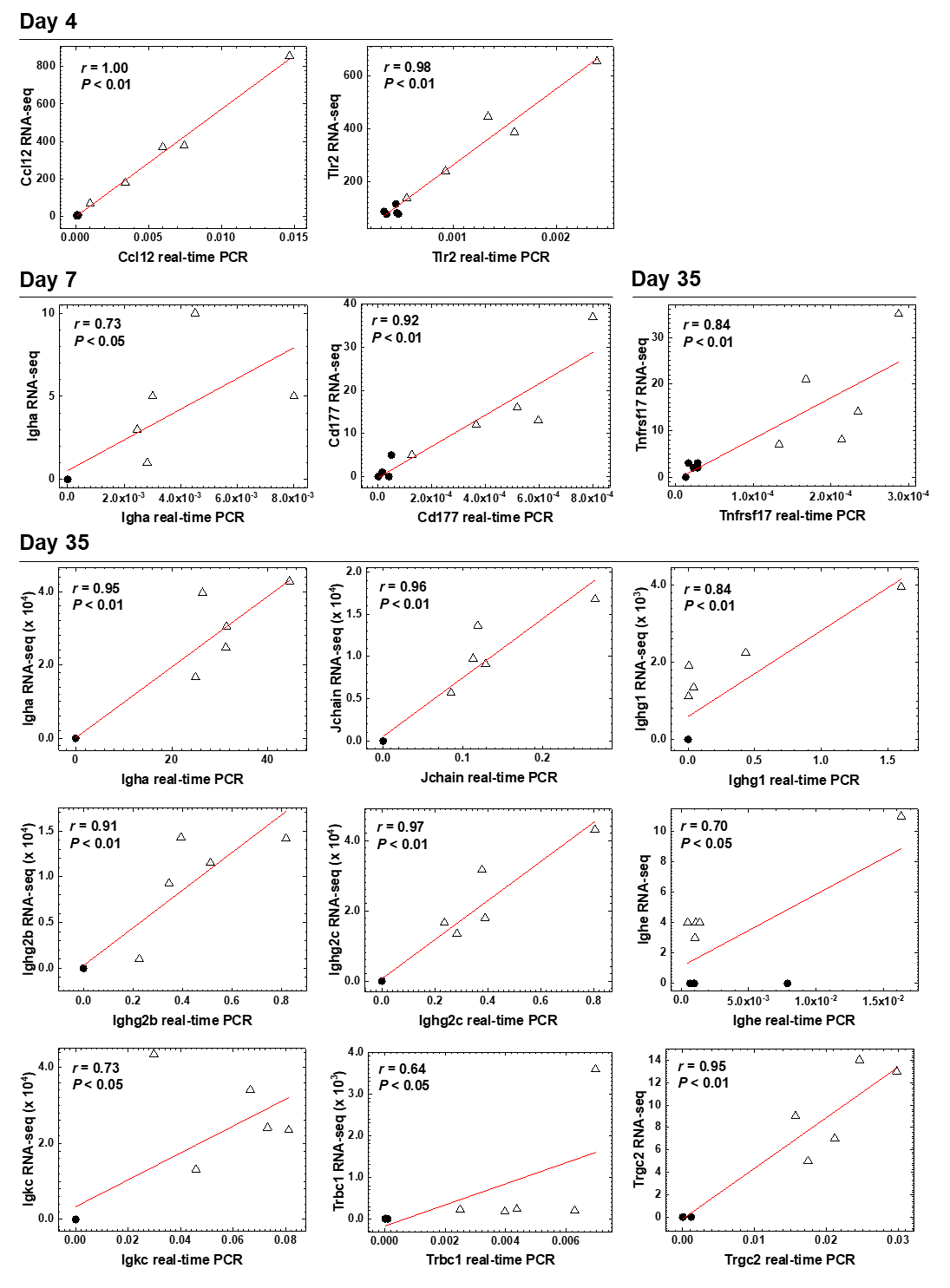
**

**Supplemental Figure 6. Validation of RNA-seq data using real-time PCR.**

Using real-time PCR, we validated the gene expression data by RNA sequencing in the spinal cord, days 4, 7, and 35 after TMEV infection. RNA sequencing data were correlated significantly with real-time PCR data at all time points. X-axis indicates the gene expressions divided by a house-keeping gene, *Gapdh*, in real-time PCR. Y-axis indicates the read count data in RNA sequencing. Abbreviations: Ccl12, chemokine (C-C motif) ligand 12; Igha, immunoglobulin (Ig) heavy constant α; Ighe, Ig heavy constant ε; Ighg1, Ig heavy constant γ 1; Ighg2, Ig heavy constant γ 2; Igkc, Ig κ constant; Tlr2, toll-like receptor 2; Tnfrsf17, tumor necrosis factor receptor superfamily, member 17 (B-cell maturation factor, BCMA, CD269); Trbc1, T cell receptor (TCR) β, constant region 1; Trgc2, TCR γ, constant 2.


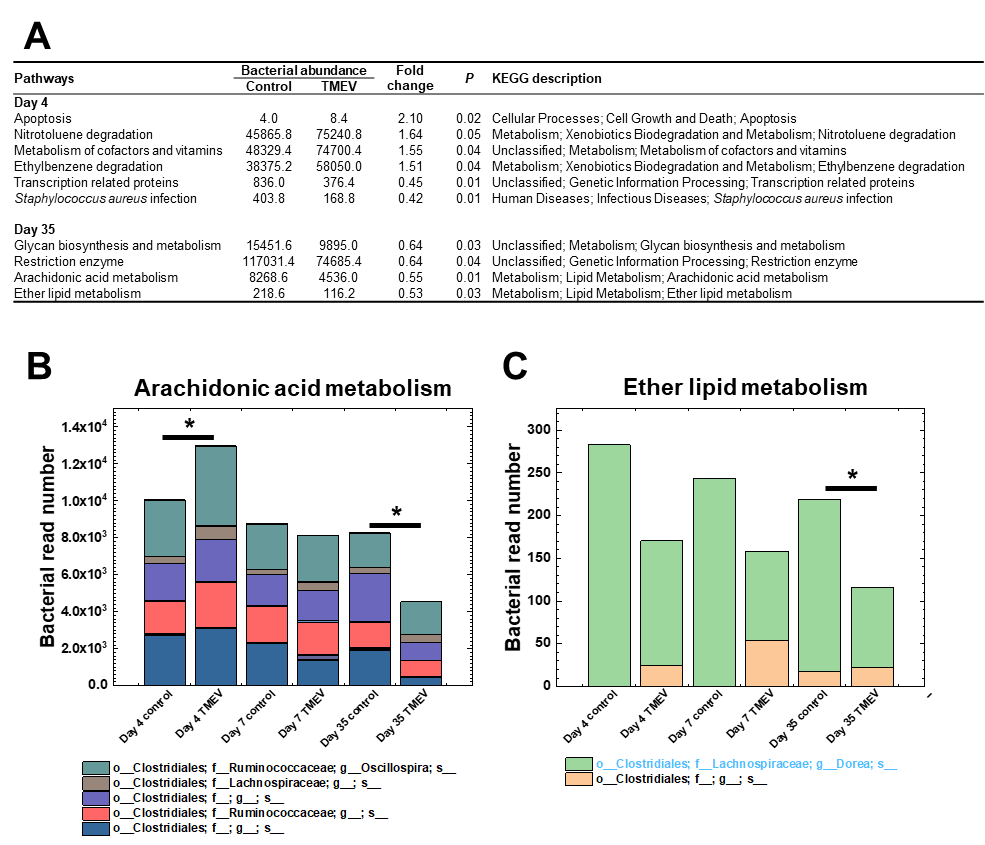


**Supplemental Figure 7. Predictive metagenome profiling using microbiome data of TMEV-infected groups by PICRUSt.**

To estimate functional changes in gut microbiome of the TMEV-infected group, we conducted a predictive metagenome analysis by PICRUSt using 16S rRNA sequencing data of fecal samples. (A) On day 4, we found significantly changes in the abundance of bacteria that have enzyme genes related to six pathways in TMEV-infected mice (Supplemental reference 1). On day 7, no significant change in bacterial functional capabilities of microbiome was predicted by PICRUSt. On day 35, the bacteria related to four pathways were significantly decreased in the TMEV-infected group. Next, among the four pathways, we identified sets of bacteria that were involved in arachidonic acid metabolism and ether lipid metabolism. (B) In the TMEV-infected groups, bacteria related to arachidonic acid metabolism were significantly increased on day 4 and decreased on day 35. The bacteria were composed of five distinct genera, all of which belong to the order *Clostridiales*. (C) On the other hand, the bacteria related to ether lipid metabolism were decreased on day 35. The bacteria were composed of two distinct genera, both of which belong to the order *Clostridiales*. *P* values were calculated by ANOVA. **P* < 0.05.

**Supplemental Table 1.** **Functional clustering of CNS transcriptome data using DAVID**

| **Annotation Cluster** | **Function** | **Enrichment score** |
| --- | --- | --- |
| **Day 4 p.i.** |  |  |
|  | Innate immune response, Immune system process | 67.05 |
|  | Defense response to virus, Response to virus | 18.48 |
|  | Cellular response to interferon-β, Defense response | 12.67 |
|  | Antigen processing and presentation of peptide antigen via MHC class I | 12.00 |
|  | Influenza A, Measles, Hepatitis C | 11.68 |
|  | Disulfide bond, Signal peptide, Integral component of membrane | 10.84 |
| **Day 7 p.i.** |  |  |
|  | Innate immune response, Immune system process | 99.57 |
|  | Disulfide bond, Signal peptide, Integral component of membrane | 39.18 |
|  | Extracellular space, Extracellular region | 17.04 |
|  | Immunoglobulin subtype, Immunoglobulin domain | 15.01 |
|  | Membrane, Integral component of membrane | 14.52 |
|  | Inflammatory bowel disease (IBD), Tuberculosis, Leishmaniasis | 14.01 |
| **Day 35 p.i.** |  |  |
|  | Innate immune response, Immune system process | 102.03 |
|  | Disulfide bond, Signal peptide, Glycoprotein | 52.77 |
|  | Cytokine-cytokine receptor interaction, Cytokine activity | 26.95 |
|  | Extracellular space, Extracellular region | 21.23 |
|  | Membrane, Integral component of membrane | 15.64 |
|  | Immunoglobulin subtype, Immunoglobulin domain | 15.58 |

p.i., post infection

To determine what kinds of genes were differentially expressed, we uploaded a list of gene IDs that showed different expression (*P* < 0.05, more than 2-fold up or downregulated between the control and treated groups) to the Database for Annotation, Visualization and Integrated Discovery (DAVID; https://david.ncifcrf.gov/). Enrichment score was calculated by Fisher’s Exact Test based on the number of differentially expressed genes in the sample, matching with the total number of genes that are included in each canonical pathway.

**Supplemental Table 5. Sequences of primer sets for real-time PCR**

| **Gene name** | **Forward primer** | **Reverse primer** |
| --- | --- | --- |
| *Ccl12* | ATTTCCACACTTCTATGCCTCCT | ATCCAGTATGGTCCTGAAGATCA |
| *Cd177* | ATACCAGTGCTGACCCTTCTG | CCTCGCAGGTTTTCTCACCA |
| *Gapdh* | CTGGAGAAACCTGCCAAGTA | TGTTGCTGTAGCCGTATTCA |
| *Igha* | TGCACAGTTACCCATCCTGA | GCACCAGCACTTCTTTAGGG |
| *Ighe* | ACACTCACCTGCTTGATCCA | TCATGGATCACTTGGCAGGT |
| *Ighg1* | CCAGACAATGTAAAGGGCCG | TAGTTTGGGCAGCAGATCCA |
| *Ighg2b* | TGGACCATCCGTCTTCATCTTC | TTCACAAACCAGCTGATCCG |
| *Ighg2c* | ACAAGAACACCGCAACAGTC | TGAGCAGGCGAAAAGACTTC |
| *Igkc* | ACCCATCGTCAAGAGCTTCA | CAAGGAAAGGGAGGAGGAGG |
| *Jchain* | TGACGACGAAGCGACCATTC | TTCAAAGGGACAACAATTCGGA |
| *Tlr2* | TCTAAAGTCGATCCGCGACAT | CTACGGGCAGTGGTGAAAACT |
| *Tnfrsf17* | GCGCAACAGTGTTTCCACAG | CGCTTGGATCACAGTAAGGCT |
| *Trbc1* | TCATGAAGCCAGATGTGAAGATG | CACCTATGCGTGACTAGTAGGC |
| *Trgc2* | ATGGGGAAAGAGCACAGTTG | AACTGGAACTGCAGCACATC |

Sequences of primer sets for *Ccl2*, *Cd177*, *Gapdh*, *Jchain*, *Tlr2*, and *Tnfrsf17* were selected from PrimerBank (https://pga.mgh.harvard.edu/primerbank/index.html). To design the primer sets for immunoglobulins and T cell receptors, we used cDNA sequences in Ensembl database (http://asia.ensembl.org/index.html) and Primer3Plus website (https://primer3plus.com/cgi-bin/dev/primer3plus.cgi).

We conducted real-time PCR to validate RNA-seq data. Total RNA was reverse-transcribed to cDNA by SuperScriptTM II Reverse Transcriptase (Thermo Fisher Scientific). Fifty ng of cDNA was applied for real-time PCR, using StepOnePlusTM real-time PCR system (Thermo Fisher Scientific) and Thunderbird^®^ qPCR Mix (TOYOBO, Osaka, Japan). *Gapdh* was used as a house-keeping gene. Correlation coefficient between the gene expression divided by Gapdh and RNA-seq data was calculated using OriginPro 2018b (OriginLab Corporation, Northampton, MA).

**Supplemental Methods**

**Predictive metagenome analysis.** We used PICRUSt (phylogenetic investigation of communities by reconstruction of unobserved states), a computational approach described by Langille et al. (Supplemental reference 2) to predict the functional composition of a metagenome of microbiota. Using 16S rRNA sequencing data of fecal microbiota by PICRUSt, we obtained two data files:

1) pathway prediction data is pathway-base and contain a list of a) the pathway name, b) the total read count data of bacteria encoding genes related to each pathway, and c) description about each pathway;

**(b)**

**(c)**

**(a)**

2) metagenome prediction data is enzyme-base and contain a list of d) the enzyme ID number, e) the total read count data of all bacteria encoding the enzyme, and f) enzyme name.

**(d)**

**(f)**

**(e)**

Using pathway prediction data, we compared the read counts between the TMEV-infected and control groups and identified the pathways with significant differences (*P* < 0.05).

Since a pathway list in pathway prediction data was based on KEGG pathway database, we obtained a list of the enzyme IDs, which was included in the identified pathway, from an ortholog table of the pathway in the KEGG pathway database (https://www.genome.jp/kegg/pathway.html) (red square in the following table).

Example) Arachidonic acid metabolism

(https://www.genome.jp/kegg-bin/show_pathway?map=map00590&show_description=show)


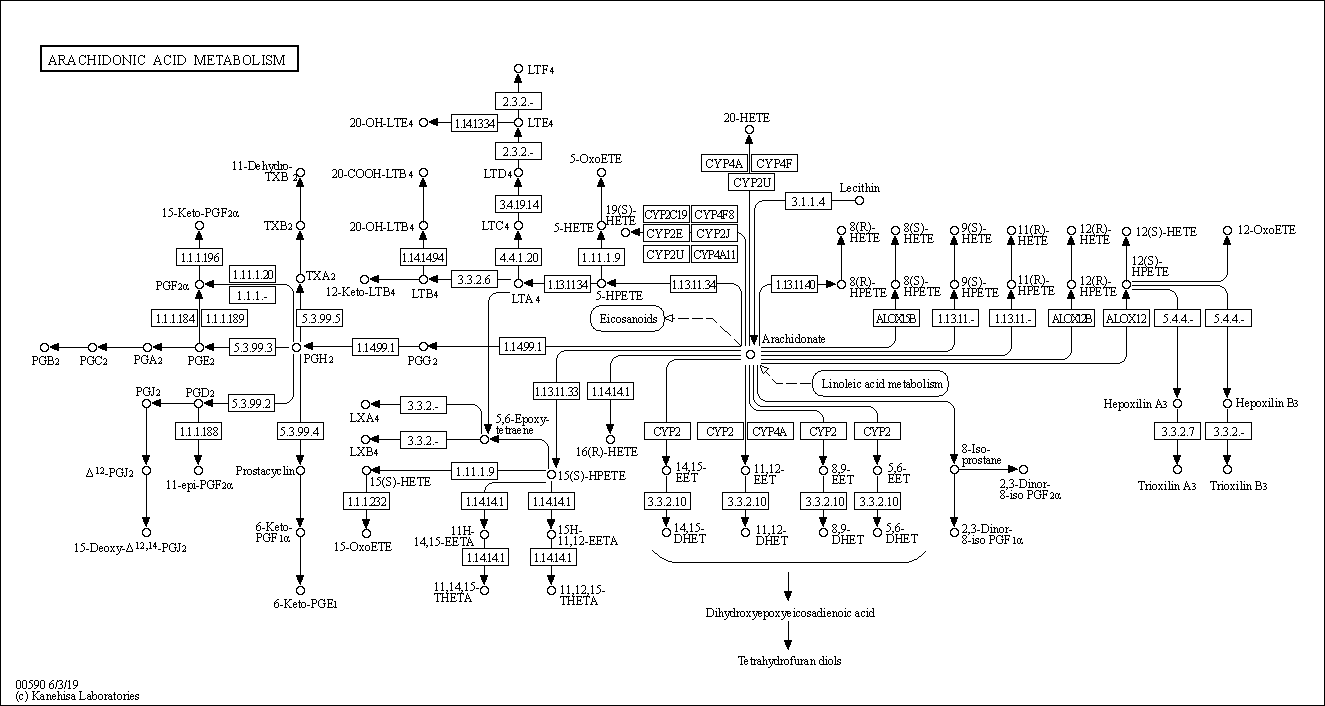


Ortholog table (https://www.kegg.jp/kegg-bin/view_ortholog_table?map=00590)


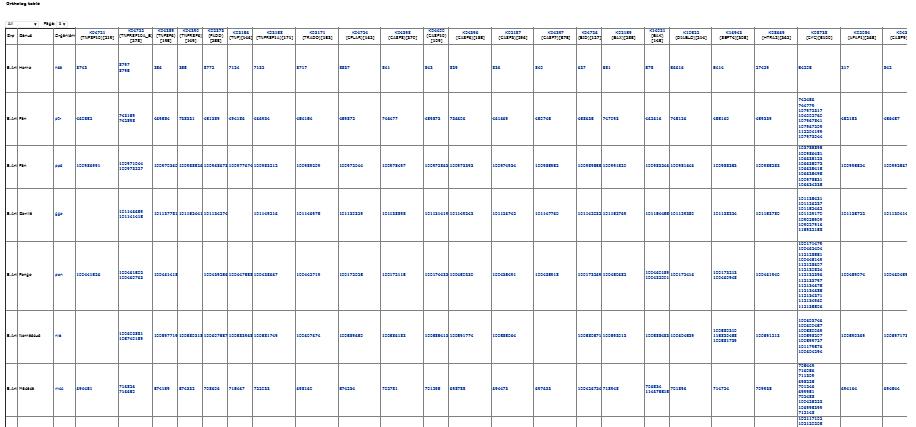


We examined whether the obtained enzyme IDs were contained in metagenome prediction data and compiled the bacterial read count data of the enzyme IDs which existed in metagenome prediction data.

Using the list of enzyme IDs, we obtained metagenome contribution data which contain the list of bacterial genera encoding the genes listed on the pathways of interest. Then, we opened the metagenome contribution data file with Microsoft Excel, sorted the data by bacterial OTU IDs (blue square in the following table) and compared the OTU IDs with Greengenes database to retrieve the information of bacterial classification corresponding to each OTU ID.

Lastly, we sorted the data by the enzymes of interest for each sample, summed the bacterial read numbers for each bacterial genus and each sample (as the following table), and drew a graph. The bacterial read number was shown per fecal sample containing 1.8 x 10^-7^ pg bacterial DNA.

The bacteria related to arachidonic acid metabolism consisted of five genera that belong to the order *Clostridiales*. Those related to ether lipid metabolism also consisted of two distinct genera that belong to the order *Clostridiales*. Here, at the genus level, each bacterial genus related to the two pathways did not show statistical changes in abundance: at the order level, there was also no significant changes in abundance of the order *Clostridiales* **(Supplemental Figure 5**). However, when we combined the bacterial numbers of the five bacterial taxa involved in arachidonic acid metabolism (**Supplemental Figure 7B**) as well as the those of the two bacterial taxa involved in ether lipid metabolism (**Supplemental Figure 7C**), we found significant differences in relative abundance of these groups of bacteria, compared with controls. Thus, neither a single bacterial genus nor bacterial order, but a set of bacteria composed of two to five genera, may play a role in metabolic changes and/or chronic phase of TMEV infection.

**Supplemental References**

1. Nagamalleswari E, Rao S, Vasu K, Nagaraja V. Restriction endonuclease triggered bacterial apoptosis as a mechanism for long time survival. *Nucleic Acids Res* (2017) **45**:8423–8434. doi:10.1093/nar/gkx576

2. Langille MGI, Zaneveld J, Caporaso JG, McDonald D, Knights D, Reyes JA, Clemente JC, Burkepile DE, Vega Thurber RL, Knight R, et al. Predictive functional profiling of microbial communities using 16S rRNA marker gene sequences. *Nat Biotechnol* (2013) **31**:814–821. doi:10.1038/nbt.2676
